# Electron attachment to CH_3_COCl molecule and clusters†

**DOI:** 10.1039/d5ra02679b

**Published:** 2025-07-10

**Authors:** Barbora Kocábková, Jozef Ďurana, Jozef Rakovský, Viktoriya Poterya, Michal Fárník, Michael Gatt, Gabriel Schöpfer, Philipp Jung, Milan Ončák

**Affiliations:** a J. Heyrovský Institute of Physical Chemistry, v.v.i., The Czech Academy of Sciences Dolejškova 2155/3 182 23 Prague Czech Republic michal.farnik@jh-inst.cas.cz; b Institut für Ionenphysik und Angewandte Physik, Universität Innsbruck Technikerstraße 25 6020 Innsbruck Austria Milan.Oncak@uibk.ac.at

## Abstract

We investigate the electron attachment of acetyl chloride CH_3_COCl (AC) molecules and clusters in a molecular beam experiment and by extensive theoretical calculations. The main product of dissociative electron attachment (DEA) to the AC molecule is Cl^−^, which leads to the main (AC)_*n*_Cl^−^ series in clusters. The weaker ion series identified in the cluster mass spectra correspond to (AC)_*n*_HCl_2_^−^ and hydrogen abstraction fragments [(AC)_*n*_–H]^−^, in full agreement with calculated energetics. We compare the present results for AC with previously studied trifluoroacetyl chloride CF_3_COCl (TFAC) and trichloroacetic acid CCl_3_COOH (TCA) molecules and clusters. DEA of the three isolated molecules results in the main fragment Cl^−^; however, the electron attachment to their clusters produces distinctly different cluster ions. This demonstrates that the outcomes of reactions of electrons with molecules in an environment cannot easily be predicted from the DEA of isolated molecules, and the solvent plays a key role in the process.

## Abstract 2

Electron attachment to halogenated molecules and their clusters: in acetyl chloride, trifluoroacetyl chloride, and trichloroacetic acid, single molecules yield a chlorine anion, while in molecular clusters, distinct products are formed.

## Graphical abstract

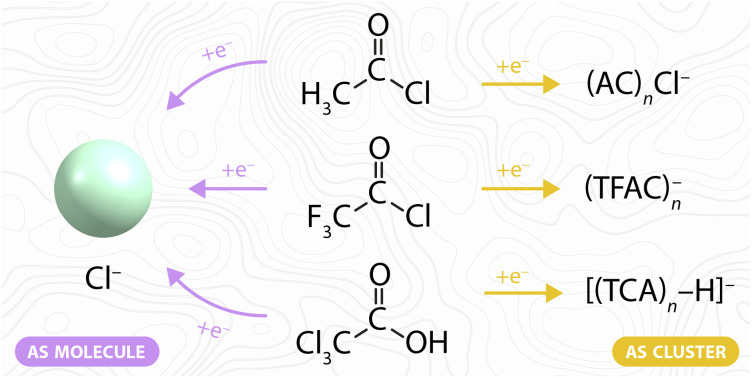

## 1. Introduction

In the context of environmental policy, chlorinated molecules are now receiving serious attention from both industry and academia. For a long time, the origin of organochlorines in the atmosphere was believed to be mainly anthropogenic, with natural sources considered to be a minor contributor. It is interesting to note that, on the one hand, some chlorinated substances have a certain degree of toxicity,^1–3^ yet, on the other hand, chlorine is commonly found in molecules present in living organisms.^4–6^ Thus, in addition to anthropogenic sources of chlorine-containing substances in the atmosphere, some of these compounds also occur naturally within the biogeochemical cycle.

For example, there is an ongoing debate regarding elevated levels of trichloroacetic acid (TCA) observed in soils.^7–9^ The proposed main mechanism for additional TCA formation begins with oxidation of C_2_Cl_4_ by a Cl radical with a reaction chain that produces trichloroacetyl chloride, which is hydrolysed to TCA in clouds.^10^ Other pathways for TCA formation have been proposed, but understanding of the atmospheric distribution and reactivity of organochlorine compounds is still lacking.^10^ Processes occurring at the air-surface interface are of increasing importance. Photoinduced surface activation, involving radical or electron chemistry with numerous intermediates, can further contribute to the atmospheric budget.

Chlorinated and fluorinated species, such as trifluoroacetyl chloride CF_3_COCl (TFAC), are formed by tropospheric photo-oxidative degradation of hydrochlorofluorocarbons,^11^ which are often used as replacements for ozone depleting chlorofluorocarbon compounds. The amount of these molecules in the atmosphere has increased in the last decades; therefore, it is important to understand their photochemistry^12^ and the processes induced by electrons.^13^

Beyond their role in atmospheric chemistry, electron-driven processes also have significant biological implications. For example, electron attachment and dissociative electron attachment are known to play a significant role in radiation damage to biomolecules, including amino acids.^14,15^ These processes can lead to the formation of reactive radicals and other species that can damage the molecular structure of biological systems.

Examples of electron activation include photosensitization reactions and solvent-induced charge delocalisation. For halogen-containing substances with high electron affinities, electron-induced reactions are another important process that contributes to the generation of reactive radicals. Research on electron attachment to molecules in different environments shows that the products of dissociative electron attachment (DEA) can be very different, going from gas-phase molecules to small clusters.^16–18^ For instance, CF_3_Cl clusters show enhancement of Cl^−^ yield at electron energies below 2 eV; this is accompanied by the dominance of new negative complex ions, such as M_*n*_Cl^−^, over spectra, suggesting an increase in autodetachment from the clusters.^13^ Halogenated organic acids, as studied in reactions with electrons, have been shown to cleave the available C–Cl bonds.^13,19^ In contrast to the predominant DEA channel in analogous organic acids, such as formic and acetic acids, where the dehydrogenation process leads to stable RCOO^−^ formation,^20,21^ the halogenated derivatives typically produce negative complexes with a halogen anion attached. Along with the hydrogen abstraction channel, stabilised undissociated negative clusters were also observed. This was confirmed for CF_3_COOH clusters, studied at energies below 2 eV by Illenberger,^22^ where the formation of undissociated negative ions (CF_3_COOH)_*n*_^−^ indicates the stabilisation effect of the cluster.

Electron attachment to halogenated acid molecules, such as trichloroacetic acid CCl_3_COOH, allows to study the competition between Cl^−^ formation and the dehydrogenation process that leads to RCOO^−^. The DEA of TCA together with CClF_2_COOH and CF_3_CHNH_2_COOH was investigated by Kopyra,^23,24^ and we have recently shown how the cluster environment influences this process.^19^

From the theoretical side, modelling of atmospherically relevant molecular clusters poses two major challenges. Firstly, for large clusters with diameters of several nanometres, it is difficult to find a suitable quantum chemical method which provides a reasonable compromise between accuracy and computational cost. This is not the main problem here, as we are considering clusters only up to pentamers. Secondly, with increasing cluster size, the number of possible isomers increases dramatically. We are already facing this challenge for our relatively small clusters with tens of atoms. Finding the energetically lowest-lying isomer is a question of global optimisation, which is a well-known problem in mathematics, engineering, computer science, and other fields.^25,26^ Global optimisation is a so-called NP-hard problem,^27^ making it particularly difficult to find general algorithms. Therefore, specialized algorithms are developed for different use cases. For molecular cluster search, popular algorithms include metaheuristic optimisation algorithms inspired by nature,^28^*e.g.*, particle swarm optimisations,^29^ the artificial bee colony algorithm,^30^ and genetic algorithms.^31^ Often, combinations or variations of these methods are being employed.^29^ Also, machine learning methods are used successfully for automated configurational sampling and efficient modelling of molecular clusters.^32^ In addition to global optimisation algorithms, rare-event molecular dynamics simulations are performed to discover reaction mechanisms and reaction networks.^33^ Here, we employ our in-house genetic algorithm^34^ which is a combination of an evolutionary genetic algorithm to cover a large portion of the potential energy surface, and gradient-driven local optimisations for refinement. By using our structure clustering algorithm to divide our population into groups of similar structures and then performing selection based on this process, we increase genetic diversity within our population, and therefore enable a more efficient search of the chemical space.

In the present study, we extend previous electron attachment studies of halogenated species^13,19^ to acetyl chloride CH_3_COCl (AC) molecules and clusters. The formation of negative ions from AC was investigated in an early mass spectrometry experiment with a trochoidal electron monochromator.^35^ We compare the present results on the AC molecule with those from previously investigated TFAC and TCA. The DEA of all three molecules results in the main fragment Cl^−^; however, the electron attachment to their clusters produces distinctly different cluster ions. This demonstrates that the outcomes of reactions of electrons with molecules in a solvent environment cannot be easily deduced even from detailed knowledge of the DEA process with isolated molecules, in agreement with earlier investigations of DEA in clusters.^18,36^

## 2. Methods

### 2.1. Experiment

The experiment was carried out using the cluster-beam (CLUB) apparatus in Prague described in detail elsewhere.^28,37^ The CH_3_COCl (Sigma-Aldrich, 99% pur.) was evaporated in a custom-built reservoir at laboratory temperature (25 °C) to a stream of buffer gas at a stagnation pressure of 1 bar. The beam was created by supersonic expansion through a conical nozzle (90 μm pinhole diameter, 30° opening, 2 mm length). Helium was used as the buffer gas for the molecular beam; clusters were obtained by co-expansion with argon as the buffer.^38^

The beam passed through a 1 mm skimmer located 18 mm downstream from the nozzle and continued through several differentially pumped vacuum chambers to the reflectron time-of-flight mass spectrometer. After a flight path of 1.5 m, the beam was ionised with a 1 μs pulse of low energy electrons (0–10 eV) from a heated tungsten filament. The detector was used in negative ion mode. Following a 0.1 μs delay (to remove any free electrons), ions were extracted by applying ±2 kV to repeller/extractor electrodes for 1 μs and further accelerated by 8 kV to the reflectron. The ions travelled approximately 95 cm in the reflectron region and were then detected with a microchannel plate.

To obtain the electron-energy-dependent ion yield, we measured the negative ion mass spectra at different electron energies scanned from 0 eV to 10 eV in steps of 0.5 eV which corresponds to our energy resolution. The ion yields were normalised by the electron current measured on a Faraday cup placed behind the ionisation region. Due to space charge and stray field effects, the measured current is expected to be lower than the actual number of electrons passing through the interaction region, and the electron energy calibration is not reliable at low energies below 2 eV. Therefore, we present here our spectra measured at the electron energies above 2 eV, as discussed also in our previous studies.^39,40^

The actual measured negative ion yields exhibit a maximum at lower electron energies and the actual resonances lay presumably close to zero, where, however, our data are not reliable. Nevertheless, the relative intensities of the reported fragments do not change significantly below 2 eV, and we assume that the electron beam at low energies contains also a significant number of near-zero energy electrons. Therefore, we assume that our low-energy spectra reflect to some extent also the resonances close to zero.

While the measured ion yields at low electron energies are underestimated due to the electron beam spread, the normalisation of the ion yield using the measured electron current overestimates the ion yields at low energies. Therefore, we show both the actually measured and the normalised ion-yield curves. These two values represent upper and lower boundaries, respectively, and the range between them corresponds to a conservative estimate of the error bars. We draw a line corresponding to average value. At higher energies, the measured and normalised values converge, since the total electron current is focused and measured on the Faraday cup.

The observed negative ions exhibit a maximum yield at low electron energies, presumably below 2 eV. Therefore, we present here the mass spectra recorded at a constant electron energy of 2 eV, where our ion signals are maximal, and the energy-dependent ion yields of the main ions are displayed in the range of 2–10 eV.

### 2.2. Calculations

For electron attachment to clusters consisting of multiple AC molecules, we employed our in-house genetic algorithm (GA),^34^ which has already been used successfully to find isomers of cluster molecules such as trichloroacetic acid clusters, hydrated silver ions, and C_120_^+^.^19,41,42^ Here, we extend the GA by natively integrating our graph-based structure clustering algorithm to enhance genetic diversity and originality of our structures. In each GA cycle, we divide our population into groups of similar structures and select the fittest structures based on their energy and group affiliation, obtaining an optimal balance between genetic diversity and energy-based fitness.

For electron attachment to a single AC molecule, we manually searched the chemical space due to the limited number of chemically meaningful possibilities.

We optimised all molecules and ions using density functional theory (DFT) at the ωB97XD/def2TZVP level of theory^43^ with tight convergence criteria to obtain zero-point energy corrected reaction energies. Furthermore, we performed single-point recalculations at the coupled-cluster level, CCSD(T)/def2TZVP, to provide more reliable energies for reactions involving smaller clusters, as well as a rough estimate of the computational error of our DFT calculations. All DFT and coupled cluster calculations were performed using Gaussian 16.^44^ The Cartesian coordinates of all optimised molecules and ions, along with their zero-point corrected electronic energies, are provided in the ESI.†

#### 2.2.1. Genetic algorithm

At the beginning of the GA, we create an initial population by randomly placing the building blocks of the respective structure in a cuboid box, *e.g.*, for (AC)_2_Cl^−^, we place two AC molecules and one chlorine atom in a box of 16 × 16 × 16 Å^3^, and set the total charge to −1. This process is repeated to generate the desired number of structures; *e.g.*, for (AC)_2_Cl^−^, we produced an initial population of 1000 structures. Generally, larger systems with more degrees of freedom require larger initial populations. Our present GA parameters were set based on our previous experience^19^ and benchmarking on the current system.

Using the semi-empirical GFN2-xTB method^45^ from the xTB software package,^46^ we optimised the neutral or anionic structures and obtained their energies and nuclear repulsion energies. Our benchmark tests suggest poor parallelisability for our comparably small systems. Therefore, we use Python's multiprocessing approach to calculate multiple structures in parallel, each on a separate core.

**Fig. 1:**
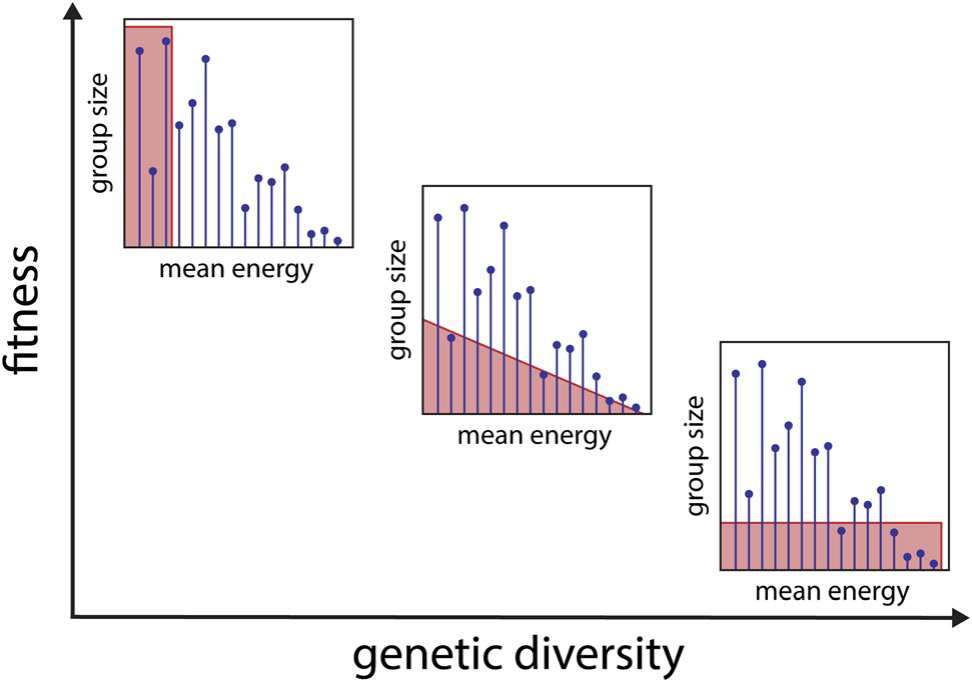
Illustration of the clustering-based selection process. After optimisation and clustering (*i.e.*, dividing the population into groups of similar structures), the selection of structures to survive until the next cycle of the genetic algorithm is performed. Each of the three insets shows the group size (number of structures in each group) as a function of their mean energy. The goal is to find an ideal balance (centre plot) between selecting the energetically fittest structures and maximizing genetic diversity rather than selecting only the fittest ones (left plot) or not regarding the energy as a criterion at all (right plot).

During the GA selection step, the structure clustering algorithm divides the population into groups (clusters) of similar structures. The number of structures to be selected from each group is based on the median energy of the respective group, as illustrated in Fig. 1. This process ensures a balance between selecting the fittest (*i.e.*, energetically lowest) structures and maintaining genetic diversity (*i.e.*, taking structures from many different groups). For example, for (AC)_2_Cl^−^, in the first cycle, 100 structures from 627 different groups were selected to form the population for the next GA cycle.

Next, a specified number of crossover operations, *e.g.*, 100 for (AC)_2_Cl^−^, is performed which, respectively, creates one new structure out of two randomly picked parent structures, taking a randomly sized share of atom positions of each structure. In addition, two different mutation operators are applied independently to each structure with a probability of 20%. The mutation functions alter the structures either by swapping the positions of two randomly chosen atoms/fragments or by randomly moving an atom/fragment. During crossover and mutation, the intermolecular distance was restricted to be no less than 1.2 Å, preventing the generation of structures with electronic wave functions that are difficult to converge. Fragments such as AC, AC^−^, or CH_3_CO are marked during initialisation of the population to keep them intact throughout crossover and mutation. This restricts the chemical space, preventing us from obtaining kinetically inaccessible structures, reflecting the limited structural rearrangement under the experimental conditions. For example, during the position switch, the position of the entire fragment is changed rather than that of an individual atom of the fragment.

**Fig. 2:**
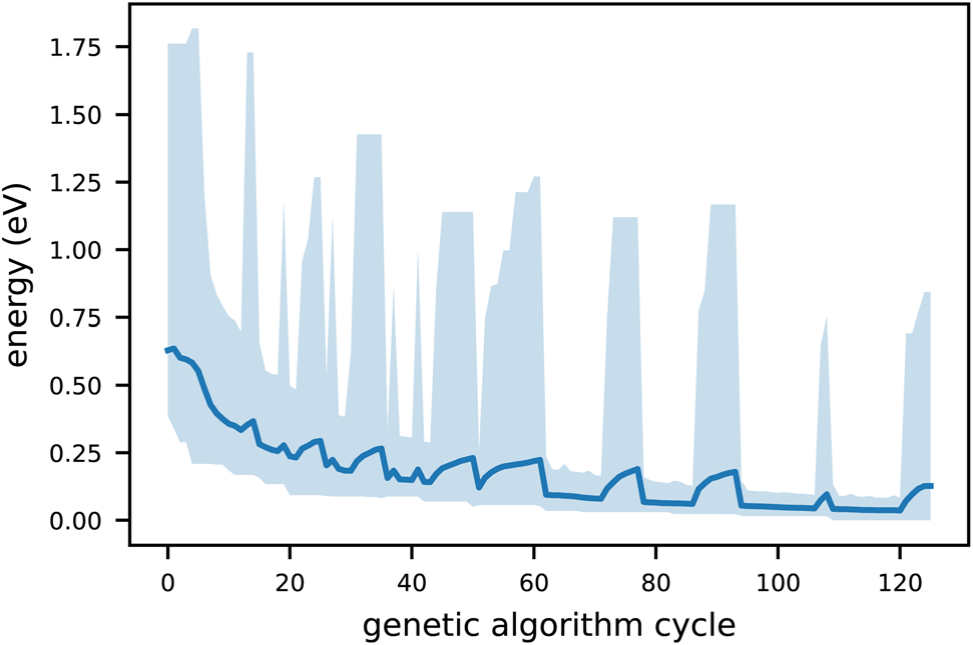
Convergence behaviour of our genetic algorithm for [(AC)_4_–H]^−^. The energy is obtained from semi-empirical optimisation using GFN2-xTB. The light blue shaded region represents the range between the minimum and maximum energy values around the mean (dark blue line).

This entire procedure is repeated for a minimum of 25 genetic cycles, depending on the cluster complexity. Fig. 2 illustrates the convergence behaviour of the GA procedure. It can be seen that the minimum energy steadily decreases, whereas the spikes in the mean and maximum energy are not adverse but simply indicate that crossover/mutation operations produced high-energy structures, further increasing the genetic diversity. All relevant parameters for the genetic algorithm runs are detailed in Table S1 (ESI).†

After completion of the GA, a final clustering of all emerged structures is performed to group similar ones. Next, all structures with an electronic energy difference of less than 0.01 eV and a nuclear repulsion energy difference of less than 0.5 hartree are considered duplicates and removed. From each group, typically three to ten of the lowest-lying or most interesting representatives are selected, depending on the size and complexity of the system. The selected candidates are then manually reviewed and, in some cases, modified based on chemical intuition before optimisation at the ωB97XD/def2TZVP level to identify minimum-energy structures. For large candidate sets, an additional intermediate optimisation at the BLYP-D3/6-31G* level helped reducing the computational cost.

#### 2.2.2. Structure clustering

Structure clustering plays a key role both for GA cycles and post-processing. Our in-house graph-based clustering algorithm creates a graph for each structure to describe its connectivity by evaluating the distance between all pairs of atoms. In this text, we use the term “group” instead of “cluster” to avoid any confusion with molecular clusters. Atom pairs are considered connected if their distances are greater than 0.8 Å and less than the respective values in the distance matrix (see Table 1). The graph is undirected, and its vertex labels represent the atomic elements.

**Table 1:** Distance matrix used within the structure clustering algorithm. Atom pairs are considered connected if their distances are greater than 0.8 Å and less than the corresponding value in the matrix. The values are derived from the distance rule *r*_*i*_ + *r*_*j*_ + 0.4 Å, where *r*_*i*_ and *r*_*j*_ denote the covalent radii of atoms *i* and *j*.^47^ Values marked with an asterisk (*) indicate manual adjustments to the originally proposed values, *e.g.*, we have allowed the O–H distance to extend up to 2.3 Å to account for hydrogen bonds. All distances are given in Å

	H	C	O	Cl
H	1.24			
C	1.5	1.76		
O	2.3 (*)	1.76	1.76	
Cl	2.8 (*)	3 (*)	3 (*)	2.38

**Fig. 3:**
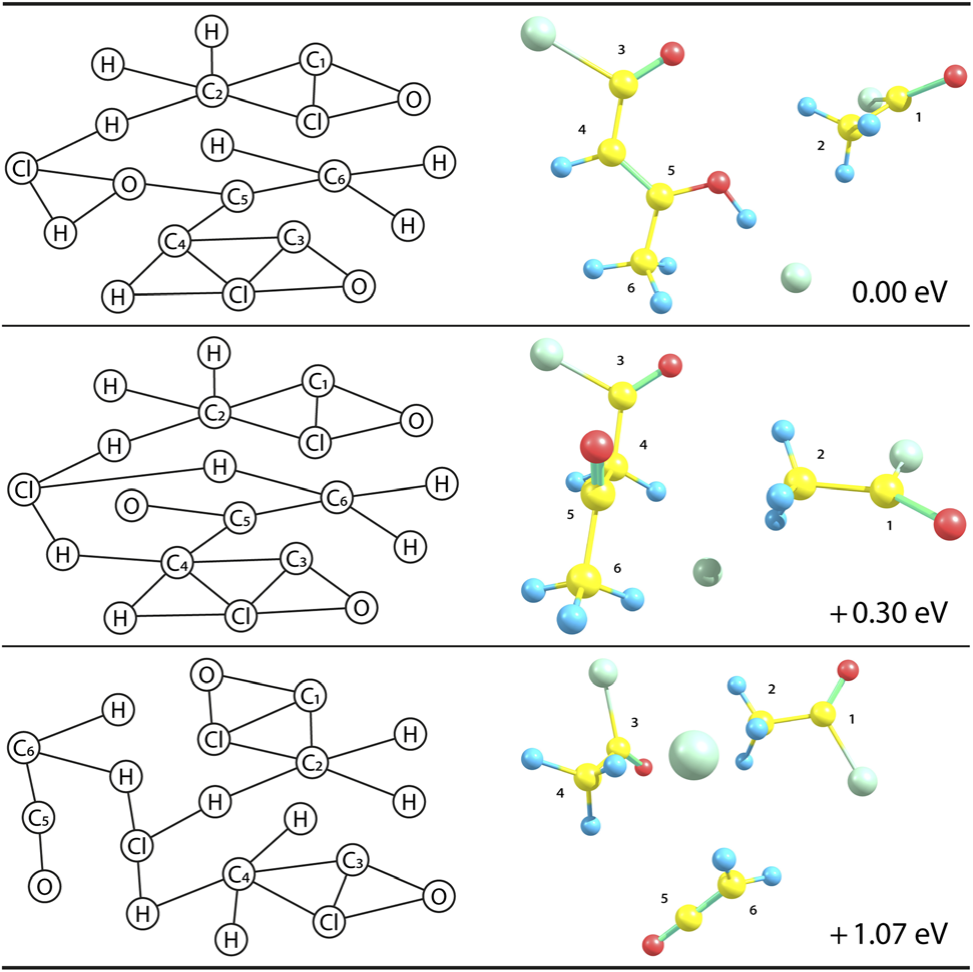
Structure clustering of an exemplary [(AC)_3_–H]^−^ population showing one representative structure from three different groups optimised at the ωB97XD/def2TZVP level (right), together with its respective graph representation (left). Note that the connecting lines in the graphs are determined by the distance thresholds as given in Table 1. The energies are reported relative to the lowest-lying structure shown in the first row. 3D structures (right) are shown in perspective, *i.e.*, closer objects appear larger.

Subsequently, groups are constructed by identifying all graphs that are isomorphic to each other. We use a backtracking algorithm from Boost Graph Library^48^ for this purpose. The worst-case time complexity of this implementation is 
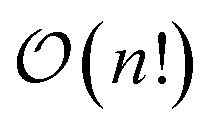
, where *n* expresses the number of graph vertices, *i.e.*, the number of atoms in the structure. Isomorphism is tested for each pair of *N* structures with 
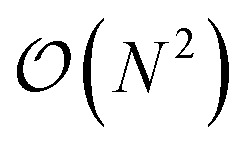
. Despite this unpleasant worst-case scaling of 
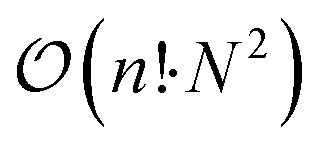
, the absolute time is only on the order of seconds to cluster thousands of structures. Typically, we only cluster fully connected graphs, since we are mostly not interested in broken structures. Fig. 3 shows example structures of [(AC)_3_–H]^−^ and their corresponding graph representation.

The structure clustering algorithm, implemented in C++ for improved performance, is integrated into the main GA code using a Python wrapper (pybind11), and available from a GitHub repository.^49^ An additional stand-alone command-line version allows users to cluster structure lists from conventional XYZ file formats. Custom covalent radii, specific pair distances, and other parameters can be defined *via* a configuration file.

## 3. Results

### 3.1. Experimental results

**Fig. 4:**
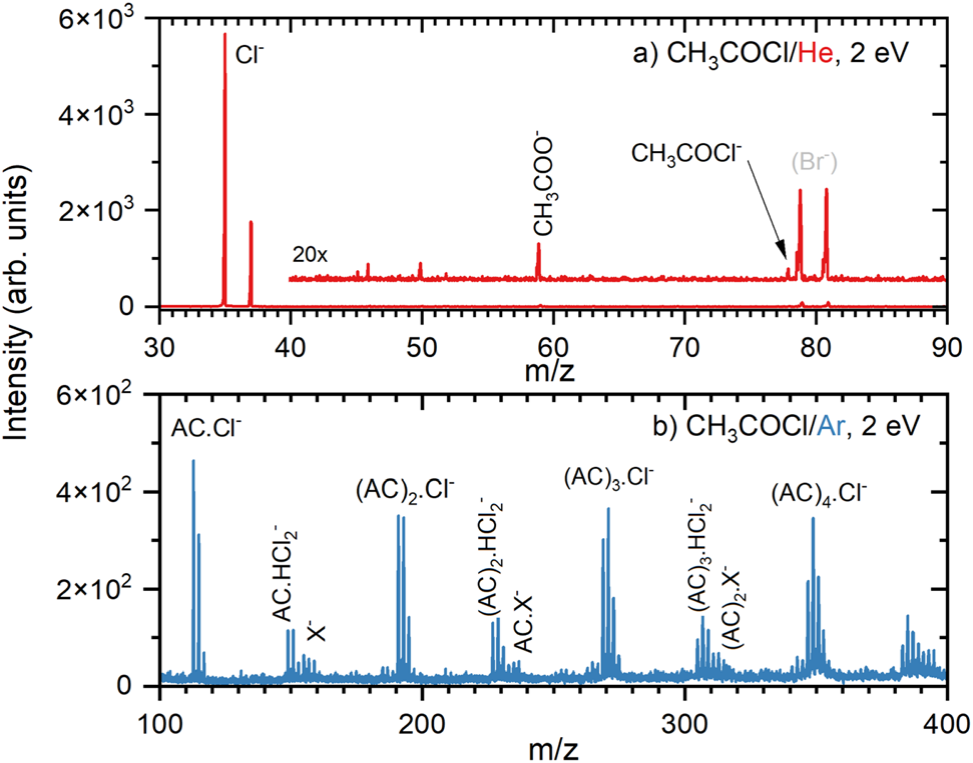
Negative ion mass spectra after the electron attachment to AC in co-expansion with He (a), and with Ar (b) at 2 eV electron energy. The possible nature of the X^−^ species is discussed in the text.

First, we briefly introduce our experimental results and then theoretically substantiate the observed anions. The negative ion mass spectrum of the AC molecule is shown in Fig. 4a. The DEA of AC yields only the Cl^−^ fragment. The 20× magnified trace exhibits Br^−^ peaks at *m*/*z* = 79 and 81 in the background of the TOF chamber due to previous long-term experiments with HBr. Only two minor peaks could not be assigned to the DEA of the monomer at the given electron energy of 2 eV and have been attributed to the presence of a small amount of (AC)_2_ dimers: *m*/*z* = 59 corresponding to CH_3_COO^−^ and a minuscule signal at *m*/*z* = 78 probably due to the parent CH_3_COCl^−^ which also originates from the dimer. The positive ion spectrum confirmed a small amount of (AC)_2_ dimers in the beam.

The clusters of AC molecules were generated in co-expansion with argon. Below the monomer mass, the cluster spectrum (Fig. S1 in ESI†) has a weaker signal, but it is essentially identical to the monomer spectrum (Fig. 4a). Above the monomer mass, the spectrum is dominated by the (AC)_*n*_Cl^−^ cluster ions, Fig. 4b. The (AC)_*n*_Cl^−^ peak multiplets correspond to isotopologues due to the natural abundance of ^35^Cl and ^37^Cl isotopes and their ratio confirms the assignment. Other fragment ion series can be assigned to (AC)_*n*_HCl_2_^−^ multiplets overlapping with other weaker ions at higher masses. The first peak of the (AC)HCl_2_^−^ multiplet at *m*/*z* = 149 is followed by two other peaks with Δ*m*/*z* = 2 due to the ^35^Cl and ^37^Cl isotopologues in intensity ratio corresponding to the natural abundance of the isotopes. There are some weak peaks between the (AC)_1_HCl_2_^−^ peaks spaced by Δ*m*/*z* = 1 from them, possibly corresponding to (AC)Cl_2_^−^.

The following smaller multiplets starting at *m*/*z* = 155 labelled X^−^ in Fig. 4b cannot be unambiguously assigned. The starting mass can correspond to the dimer after H abstraction [(AC)_2_–H]^−^. This ion contains 2 Cl atoms, which would yield the isotopologue abundance at *m*/*z* = 159 of about 10% of the main peak at *m*/*z* = 155. In the spectrum, the peak at *m*/*z* = 159 accounts for almost 80% of *m*/*z* = 155. Thus, we can assume that other ions contribute to *m*/*z* = 159. Possible candidates for the mass peak at *m*/*z* = 157 can be AC anions with an additional H-atom, *i.e.*, [(AC)_2_+H]^−^. Our theoretical calculations suggest that the pathway from the AC trimer anion, (AC)_3_^−^ → [(AC)_2_+H]^−^ + [AC–H], requires only about 0.50 eV energy, which is easily available in our experiment (see below). Furthermore, peaks with lower intensity at even masses starting at *m*/*z* = 156 are also observable, and their identification implies a contribution of the unfragmented AC dimer anion, but as a minor product, since the peak intensity is less than 50% of *m*/*z* = 155. Thus, we suggest that the peaks labelled X^−^ in our spectrum correspond to [(AC)_2_–H]^−^, [(AC)_2_]^−^, and [(AC)_2_+H]^−^ anions. The corresponding fit of the peak intensities assuming the natural abundances of the isotopologues is not perfect (see Fig. S4†); yet the errors in the ion abundances might be quite large, considering the low intensities of the mass peaks in this region. In addition, other anions might contribute. We could propose a few anions that fit the observed *m*/*z* simply by combining the masses of the available atoms. However, our theoretical calculations do not provide any support for the structure of these ions, and therefore we mention them only in the ESI.†

Similar analysis can be performed for the next series of peaks starting with the (AC)_2_HCl_2_^−^ multiplet at *m*/*z* = 227. Again, the peak at *m*/*z* = 233 corresponds to the H abstraction product [(AC)_3_–H]^−^, however, the ion corresponding to *m*/*z* = 237 seems to be more abundant than the corresponding isotopologue of [(AC)_3_–H]^−^. Thus, we could invoke here the [(AC)_3_+H]^−^ series starting at *m*/*z* = 235. Further minor series at higher masses above (AC)_3_ were too weak and congested to be analysed in detail.

In summary, the negative ions are composed mainly of (AC)_*n*_Cl^−^ ion series, followed by (AC)_*n*_HCl_2_^−^ (accompanied by weak (AC)_*n*_Cl_2_^−^ series). The weak mass peaks labelled as (AC)_*n*_X^−^ in Fig. 4 are tentatively assigned to cluster ions with H atom subtraction and addition, *i.e.*, [(AC)_*n*_–H]^−^ and [(AC)_*n*_+H]^−^, respectively, (accompanied by weak (AC)_*n*_^−^ series). Further ions could be involved, as discussed in the SI. Nevertheless, the weak abundances and mass congestion due to isotopologues prohibit an unambiguous assignment of these weak mass peaks.

**Fig. 5:**
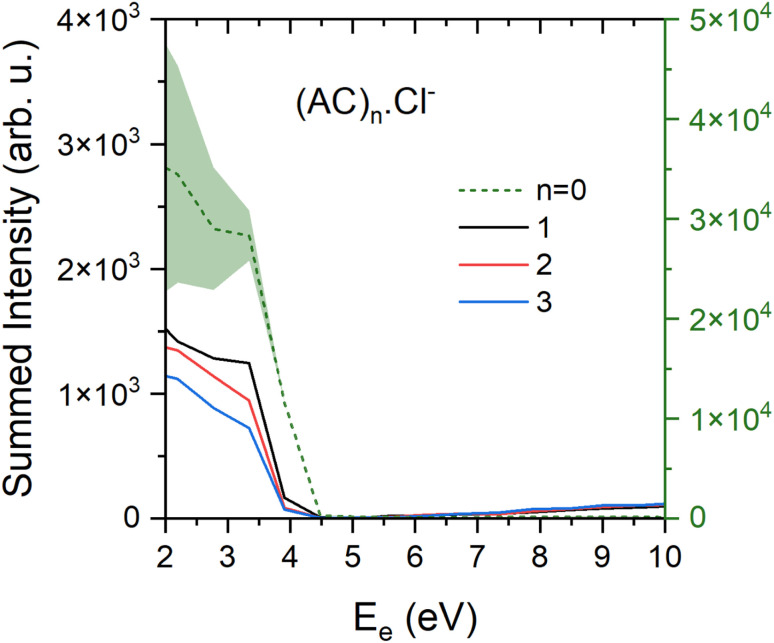
Electron energy dependent yield for the (AC)_*n*_Cl^−^ clusters for *n* = 0–3. The Cl^−^ fragment (green dashed line) corresponds to the right *y* axis, light green area represents conservative error bars for Cl^−^, which are explained in the experimental section. For *n* = 1–3 the error bars are omitted for picture clarity. They are proportionally similar for all other fragments.

The mass spectra were also measured at the higher electron energy of about 6 eV. At these higher energies, all intensities decrease significantly and only the major (AC)_*n*_Cl^−^ series is discernible above the noise (ESI, Fig. S2†). Fig. 5 shows the yield of the major (AC)_*n*_Cl^−^ fragment anions, *n* = 0–3, in dependence on the electron energy. The yields closely resemble the Cl^−^ yield of isolated AC molecules measured in He and shown in ESI, Fig. S3.† They increase from 4.5 eV towards lower energies. As described in the Experimental section, the yields below 2 eV cannot be evaluated reliably as indicated by the increasing error bars for the Cl^−^ ion. Thus, the electron attachment leading to the (AC)_*n*_Cl^−^ anions exhibits a maximum at a low electron energy below 2 eV. The slight increase in the intensity of the (AC)_*n*_Cl^−^, *n* ≥ 1, anions towards higher energies of 10 eV may be due to insufficient background subtraction and is within our error bars. The abundances of the other anion fragments apart from (AC)_*n*_Cl^−^ are too small to evaluate the energy-dependent ion yield.

### 3.2. Theoretical results

**Fig. 6:**
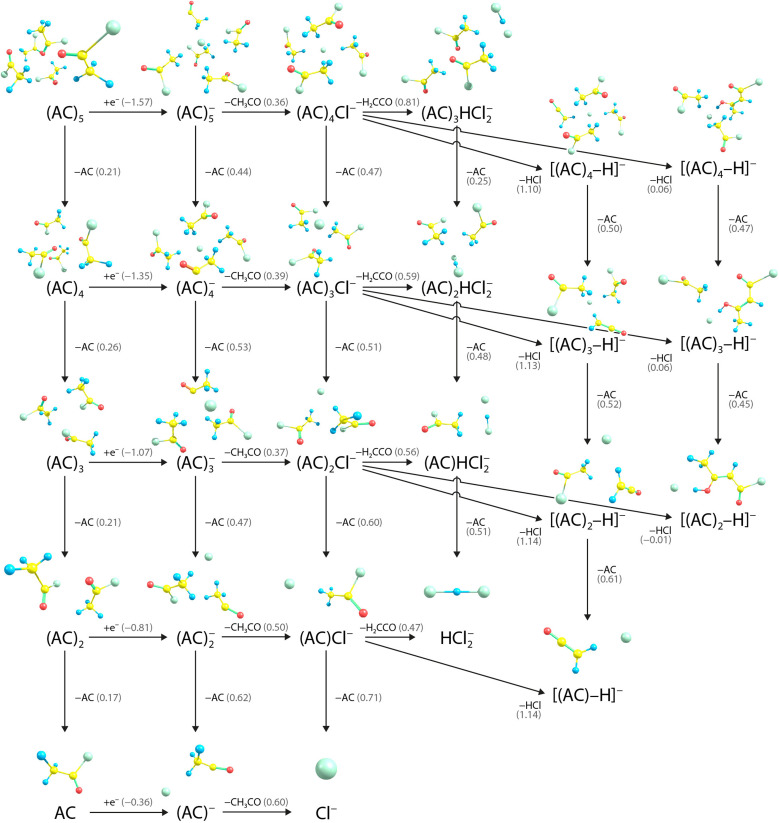
Suggested fragmentation pathways before and after electron attachment to clusters and monomers of AC as calculated at the ωB97XD/def2TZVP level of theory. Reaction energies and adiabatic electron affinities in eV are denoted in brackets.

To provide tentative structures for all observed fragments and further interpret the experimental findings, we performed calculations on the title clusters. The results of our calculations, in combination with the experimental results, allow us to suggest reaction pathways, gaining insights into the chemistry of acetyl chloride clusters. A summary of all reactions is given in Fig. 6, energetics of selected reactions are collected in Tables 2 and 3.

**Table 2:** Total energies of various reactions including monomer, dimer, and trimer forms of AC, calculated at the ωB97XD/def2TZVP level. Energies denoted in brackets are obtained with coupled cluster single-point recalculations at the CCSD(T)/def2TZVP//ωB97XD/def2TZVP level of theory

Reaction	*E* (eV)
AC + e^−^ → (AC)^−^	−0.36 (−0.15)
Cl^−^ + CH_3_CO	0.23 (0.41)
(AC)_2_ + e^−^ → (AC)_2_^−^	−0.81 (−0.54)
(AC)_2_ → AC + AC	0.17 (0.18)
(AC)_2_^−^ → (AC)Cl^−^ + CH_3_CO	0.50 (0.46)
(AC)^−^ + AC	0.62 (0.57)
(AC)_3_ + e^−^ → (AC)_3_^−^	−1.07 (−0.78)
(AC)_3_ → (AC)_2_ + AC	0.21 (0.21)
(AC)_3_^−^ → (AC)_2_Cl^−^ + CH_3_CO	0.37 (0.35)
(AC)_2_^−^ + AC	0.47 (0.46)
(AC)_2_Cl^−^ → (AC)Cl^−^ + AC	0.60 (0.57)
(AC)HCl_2_^−^ + H_2_CCO	0.56 (0.61)

**Table 3:** Selected reaction channels leading to Cl^−^ formation as calculated at the ωB97XD/def2TZVP level of theory. Reaction energies are reported in eV, with empty cells indicating reactions that require at least AC hexamers as a starting unit or are not possible due to a small cluster size

Reaction	*n* = 5	4	3	2	1
(AC)_*n*_^−^ → Cl^−^ + (AC)_*n*−1_CH_3_CO	2.00	1.69	1.36	1.08	0.60
(AC)_*n*_Cl^−^ → Cl^−^ + (AC)_*n*_	—	1.66	1.44	1.15	0.71
(AC)_*n*_HCl_2_^−^ → Cl^−^ + (AC)_*n*_HCl	—	—	1.95	2.01	1.65
[(AC)_*n*_–H]^−^ → Cl^−^ + (AC)_*n*−1_H_2_CCO	—	1.65	1.42	1.08	0.56
[(AC)_*n*_–H]^−^ (m)^a^ → Cl^−^ + (AC)_*n*−1_H_2_CCO (m)	—	2.22	1.95	1.81	—

a Merged structures are marked with (m).

Using the structures explored with our genetic algorithm, we computed the energies of reactions upon electron attachment to AC at the ωB97XD/def2TZVP level of theory. Single-point recalculations at the CCSD(T)/def2TZVP level for selected reactions confirm that DFT provides reliable results, as shown in Table 2, which justifies our reliance on DFT values for larger clusters, where coupled cluster calculations would be computationally prohibitive.

In the experiment, first, neutral AC clusters are produced. As shown in Fig. 6, losing an AC molecule from these (AC)_*n*_ clusters is endothermic by only around 0.2 eV, making it possible for the clusters to shrink even before the attachment of an electron. Therefore, any fragment observed in the experiment might be originally derived from a larger cluster. After cluster production, an electron with an energy of 2 eV is attached to the clusters. Upon electron attachment, geometry optimisation suggests that the C–Cl bond breaks, and the Cl^−^ anion migrates into the vicinity of the CH_3_ groups, with the excess electron residing at the Cl^−^ anion. This structure can be denoted as [Cl^−^(AC)_*n*_CH_3_CO], indicating that the AC molecule prevails in a non-intact form. The adiabatic electron attachment is exothermic by 0.36 eV for a single AC molecule, and increases with cluster size to 1.57 eV for (AC)_5_ due to the favourable interaction of Cl^−^ with the AC units. This leads to a total available energy in the clusters of about 2–4 eV for subsequent reactions. As CH_3_CO is not covalently bound to the rest of the cluster, the energy required for CH_3_CO to leave the cluster and produce (AC)_*n*_Cl^−^ is only 0.36–0.60 eV, depending on the cluster size. This explains why (AC)_*n*_Cl^−^ is the second most prominent peak in the experimental spectrum after the pure Cl^−^ peak.

The central role of Cl^−^ in the DEA of AC clusters can be clearly seen by the reaction pathways collected in Fig. 6. Although production of Cl^−^ requires more energy than, *e.g.*, formation of (AC)_*n*_Cl^−^, it dominates in the mass spectrum (see Fig. 4 and ESI, S1†) as it is a sink ion with many pathways ending in this ion as documented by Table 3 that lists several reactions for formation of the chlorine ion from various anions. Besides, the cluster beam probably contains quite some number of non-clustered isolated monomers that yield Cl^−^ as the main DEA product.

Looking again at the (AC)_*n*_Cl^−^ clusters in Fig. 6, we can identify three more dissociation pathways for this cluster besides loss of Cl^−^: loss of AC, H_2_CCO or HCl. The first one is the trivial loss of an AC molecule, reducing the cluster size by one AC unit. This loss is endothermic by 0.47–0.71 eV, making it easily accessible and opening another pathway to shrink the cluster size. The second pathway is arguably more interesting, describing the production of (AC)_*n*_HCl_2_^−^ by dissociation of H_2_CCO. This reaction is endothermic by only 0.47–0.81 eV, explaining why (AC)_*n*_HCl_2_^−^ is the third most prominent peak in the experimental spectrum (Fig. 4). This pathway also seems quite easily accessible from a kinetic perspective: The Cl^−^ anion in (AC)_*n*_Cl^−^ takes an H atom from the CH_3_ group, leading to the formation of a C=C double bond and subsequent C–Cl bond cleavage. The arising Cl^−^ anion then interacts with the HCl molecule, leading to the formation of (AC)_*n*_HCl_2_^−^. The resulting neutral H_2_CCO simply leaves the cluster. In our calculations, clusters including the HCl_2_^−^ moiety were found to be preferred both energetically and kinetically compared to other isomers of (AC)_*n*_HCl_2_^−^ stoichiometry. It should be mentioned here that the single HCl_2_^−^ anion has also been observed in the experiment (see ESI, Fig. S1†). The HCl_2_^−^ ion is missing in Fig. 4a as this is the spectrum of the AC monomer.

The third dissociation pathway for (AC)_*n*_Cl^−^ is the loss of HCl, producing [(AC)_*n*_–H]^−^ anions. We suggest here two qualitatively different reaction products, which are shown in the last two columns of Fig. 6. The first structure corresponds to the simple HCl dissociation without significant reorganisation inside the cluster. This reaction is kinetically and energetically easily accessible, explaining the occurrence of [(AC)_*n*_–H]^−^ in the spectrum. However, since this reaction is energetically higher than the loss of H_2_CCO, it explains why the [(AC)_*n*_–H]^−^ peaks are less abundant than the ones of (AC)_*n*_HCl_2_^−^ in the experiment. The alternative possibility for HCl loss with formation of a new carbon–carbon bond is given in the last column of Fig. 6. On the one hand, this pathway is around 1 eV lower in energy than the first one; on the other hand, it seems kinetically much harder. It is thus unclear which of the two alternative pathways is dominant in the experiment.

**Fig. 7:**
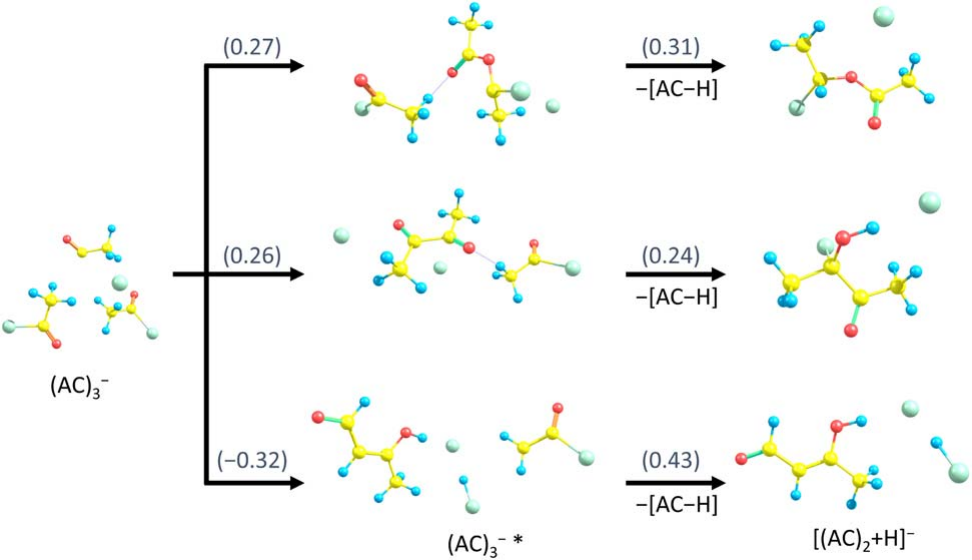
Suggested fragmentation channels from (AC)_3_^−^ to [(AC)_2_+H]^−^, with reaction energies in eV calculated at the ωB97XD/def2TZVP level of theory. The bottom structure of [(AC)_2_+H]^−^ is energetically more stable, while the two top structures are likely more easily accessible kinetically.

A similar discussion can be conducted for the experimental observation of [(AC)_*n*_+H]^−^. Possible pathways are shown in Fig. 7 for the example of [(AC)_2_+H]^−^. All pathways would be energetically accessible in the experiment. However, the upper two pathways are energetically higher, whereas the bottom pathway is kinetically more difficult to follow, leaving the uncertainty with respect to the isomer distribution of [(AC)_*n*_+H]^−^ in the experiment. Even though production of [(AC)_*n*_+H]^−^ is energetically more favourable than that of (AC)_*n*_HCl_2_^−^, we can tentatively explain the lower experimental abundance by more complicated kinetics.

Overall, our theoretical calculations match quite well with the experimental results, giving further insight into the involved structures and reaction mechanisms. The Cl^−^ anion can be identified as the driving force for basically all observed reactions.

## 4. Discussion

The electron attachment to AC molecules was previously investigated in an early mass spectrometry experiment with a trochoidal electron monochromator.^35^ Cl^−^ was produced with zero kinetic energy electrons, and the resonance extended to about 2.5 eV (with 0.2 eV resolution). However, the authors also observed other minor DEA channels, *e.g.*, CH_2_COCl^−^ at ≈0 eV, C_2_H_3_O^−^ at ≈1 eV, and O^−^ at ≈9 eV. In our present study, we observe only the Cl^−^ anion. The abundance of the other ions in our experiment is probably too low to be detected, referring to their lower signal-to-noise ratio compared to Cl^−^ as observed in ref. 35. We concentrate on the electron attachment to the clusters.

The major anion series (AC)_*n*_Cl^−^ is well substantiated by our calculations. They show that the electron attachment to (AC)_*n*_ cluster generates a transient negative ion (TNI) and the released energy leads to the change in the structure of the cluster and subsequent CH_3_CO release yielding (AC)_*n*−1_Cl^−^. These processes are energetically easily accessible. Further evaporation of H_2_CCO from the cluster anion, leading to the next weaker observed series (AC)_*n*_HCl_2_^−^, which is energetically more demanding, yet still possible at our electron energy of 2 eV. Further very weak [(AC)_*n*_–H]^−^ series originates from the H abstraction. Two different pathways are proposed. In comparison to the (AC)_*n*_HCl_2_^−^ ion formation, one channel is energetically higher, whereas the other one is kinetically less accessible, explaining the lower abundance of the [(AC)_*n*_–H]^−^ series. In addition, contributions to the [(AC)_*n*_H]^−^ series are suggested. However, the abundances of these ions in our experiment are too low for an unambiguous analysis. Nevertheless, the formation of the main (AC)_*n*_Cl^−^ ions and two minor series (AC)_*n*_HCl_2_^−^ and [(AC)_*n*_–H]^−^ observed in the experiment is well explained by our calculations.

Finally, we focus on differences and similarities of chemical patterns with respect to previous studies on similar molecules. First, we compare the electron attachment to the present acetyl chloride molecule, CH_3_COCl, with that to trichloroacetic acid (TCA, CCl_3_COOH). The electron attachment to TCA was previously investigated for isolated molecules^23,24^ and clusters.^19^ Both the AC and TCA molecules produce Cl^−^ as by far the major product upon DEA. In both cases, the cluster anion mass spectra are also dominated by a strong Cl^−^ peak at low electron energies around 2 eV. However, it is not possible to distinguish between the contribution of Cl^−^ escaping from the clusters and the Cl^−^ contribution originating from the DEA of the isolated molecules present in the beam.

The main cluster ions in the part of the AC spectrum beyond the monomer mass are the (AC)_*n*_Cl^−^ fragments. On the other hand, in (TCA)_*n*_ clusters, there are no (TCA)_*n*_Cl^−^ ions. Instead, the H abstraction channel yielding [(TCA)_*n*_–H]^−^ dominates the cluster spectrum. This demonstrates a quite different fragmentation of these molecules in an environment despite the common most abundant Cl^−^ fragment for both isolated molecules. However, it should be noted that the Cl^−^ fragment originates from C–Cl at different positions in the two molecules.

Furthermore, we can compare the present AC with the previously studied trifluoroacetyl chloride (TFAC, CF_3_COCl),^13^ where the Cl atom is in the same position and CH_3_ is replaced with the electronegative CF_3_ group. Again, Cl^−^ is the main fragment of the TFAC molecule at low electron energies around 2 eV (at higher energies above 4 eV, CF_3_^−^ dominates). Once again, TFAC fragments differ upon complexation in clusters. At low electron energies around 2 eV, essentially intact (TFAC)_*n*_^−^ ions dominate the spectrum with a small contribution of (TFAC)_*n*_Cl^−^ fragments. However, at higher electron energies above approximately 5 eV, (TFAC)_*n*_Cl^−^ become dominant and (TFAC)_*n*_Cl_2_^−^ occur, while (TFAC)_*n*_^−^ remain as minor peaks in the spectrum.

**Fig. 8:**
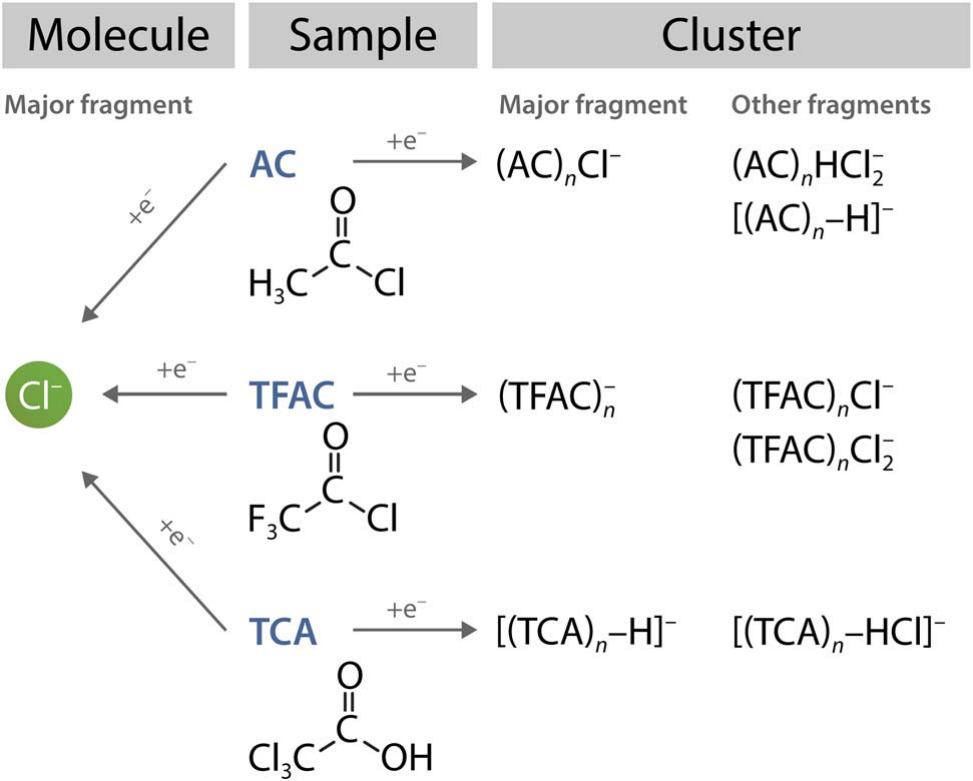
Fragmentation pathways upon attachment of 2 eV electrons to similar molecules (AC, TCA and TFAC) and their clusters. All three molecules have as isolated molecule Cl^−^ as the major product of dissociative electron attachment (left-pointing arrows), while in an environment, their fragmentation patterns are unique (arrows pointing right).

The take-home message from this comparison is that assumptions about molecular fragmentation upon DEA in an environment cannot generally be based upon the fragmentation observed for isolated molecules. Here, we have examples of three similar molecules (AC, TCA and TFAC) yielding Cl^−^ as the major DEA product, yet they all exhibit a different fragmentation pattern in clusters (at the same electron energies). This is illustrated schematically in Fig. 8.

## 5. Conclusions

We investigated the electron attachment of acetyl chloride molecules and clusters in a molecular beam experiment and by extensive theoretical calculations. The main DEA product of the AC molecule is Cl^−^, which leads to a prominent (AC)_*n*_Cl^−^ series in clusters. This is well substantiated by our calculations. Further unambiguous weaker ion series (AC)_*n*_HCl_2_^−^ and [(AC)_*n*_–H]^−^ in the mass spectra were identified and computationally justified. A possible minor contribution of other ions has been discussed. Our global optimisation effort using genetic algorithm search revealed a rich potential energy surface of the weakly bound clusters.

The main conclusion of our present investigation concerns a comparison with similar other molecules summarised in Fig. 8. The DEA of the three isolated molecules AC, TFAC, and TCA, results mainly in the Cl^−^ anion. However, the electron attachment to their clusters yields distinctly different cluster anions. This finding underlines the main message of our present study: outcomes of reactions of electrons with molecules in solvent cannot be easily predicted from the DEA process in the gas phase. The study emphasizes the significance of cluster investigations in understanding the effects of solvents at the molecular level.

## Author contributions

Barbora Kocábková: data curation (lead); formal analysis (equal); investigation (equal); visualization (equal); writing – review & editing (supporting). Jozef Ďurana: data curation (supporting); formal analysis (supporting); investigation (supporting). Jozef Rakovský: investigation (supporting); methodology (supporting); supervision (supporting). Viktoriya Poterya: investigation (supporting); methodology (supporting); supervision (supporting); formal analysis (equal); writing – review & editing (supporting). Michal Fárník: conceptualization (lead); formal analysis (equal); funding acquisition (lead); investigation (equal); methodology (equal); project administration (lead); supervision (equal); validation (lead); visualization (supporting); writing – original draft (lead); writing – review & editing (equal). Michael Gatt: data curation (lead); formal analysis (equal); investigation (equal); visualization (equal); writing – original draft (equal). Gabriel Schöpfer: formal analysis (equal); investigation (equal); writing – original draft (equal). Philipp Jung: investigation (supporting); methodology (equal). Milan Ončák: conceptualization (lead); formal analysis (equal); investigation (equal); methodology (equal); project administration (lead); supervision (equal); validation (lead); writing – review & editing (equal).

## Conflicts of interest

The authors have no conflicts to disclose.

## Supplementary Material

RA-015-D5RA02679B-s001

## Data Availability

The data that support the findings of this study are available within the article and its ESI.† Further data are available from the public repository DOI: https://doi.org/10.48700/datst.yqs1c-ekp89.
