# Supplementary material for: Electron attachment to CH3COCl molecule and clusters
Source: RSC Adv. 2025 Jul 10;15(29):23983–93. doi: 10.1039/d5ra02679b (PMC12242388; doi:10.1039/d5ra02679b)
Supplement: RA-015-D5RA02679B-s001 [file RA-015-D5RA02679B-s001.pdf]

Supplementary Information (SI) for RSC Advances.
This journal is © The Royal Society of Chemistry 2025

SUPPORTING INFORMATION FOR:

Electron attachment to CH_3COCl molecule and clusters

Barbora Kocábková,* Jozef Ďurana,* Jozef Rakovský, Viktoriya Poterya, and Michal Fárník†

J. Heyrovský Institute of Physical Chemistry, v.v.i.,

The Czech Academy of Sciences, Dolejškova 2155/3, 182 23 Prague, Czech Republic

Michael Gatt, Gabriel Schöpfer, Philipp Jung, and Milan Ončák‡

Institut für Ionenphysik und Angewandte Physik,

Universität Innsbruck, Technikerstraße 25, 6020 Innsbruck, Austria

(Dated: April 16, 2025)

I. MASS SPECTRA

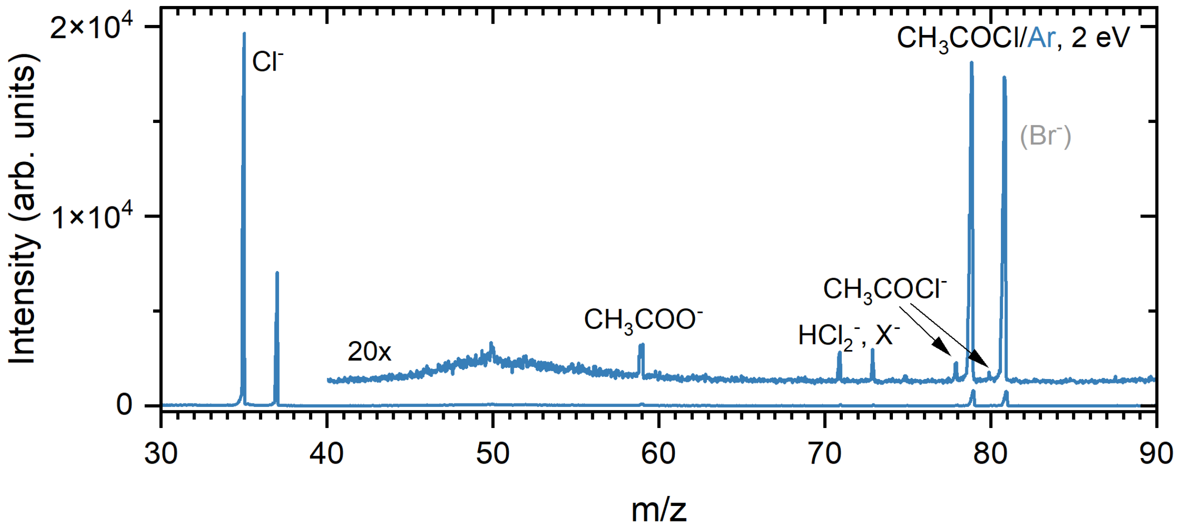

Fig. S1. The mass spectrum of AC co-expanded in Ar within the molecule ($m/z = 78$) mass range at 2 eV electron energy.

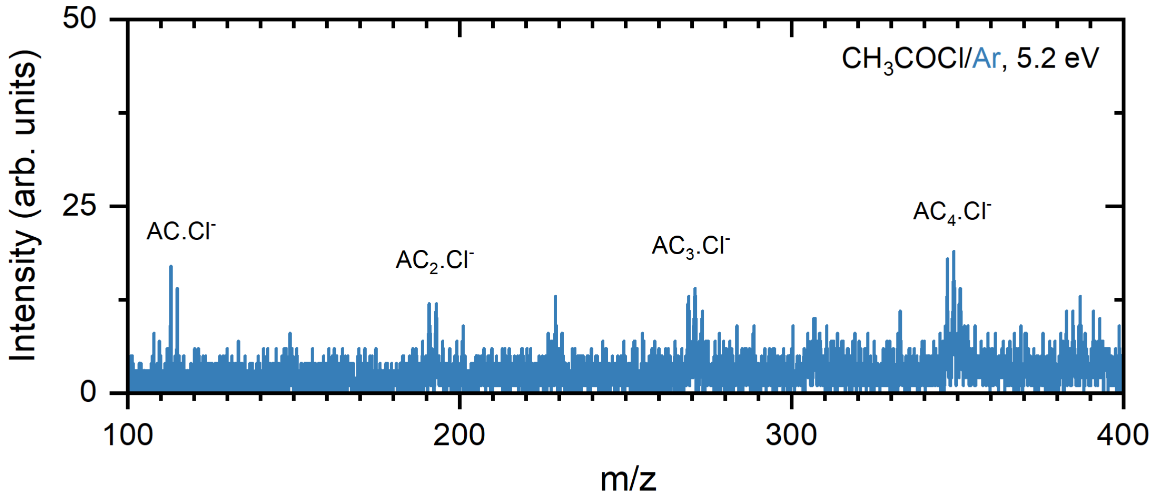

Fig. S2. Spectrum of AC clusters (co-expansion in Ar) at around 6 eV.

* Also at: Institute of Physical Chemistry, University of Chemistry and Technology, Technická 5, Prague 6, 166 28, Czech Republic

† michal.farnik@jh-inst.cas.cz

‡ Milan.Oncak@uibk.ac.at

II. ELECTRON ENERGY DEPENDENT ION YIELD

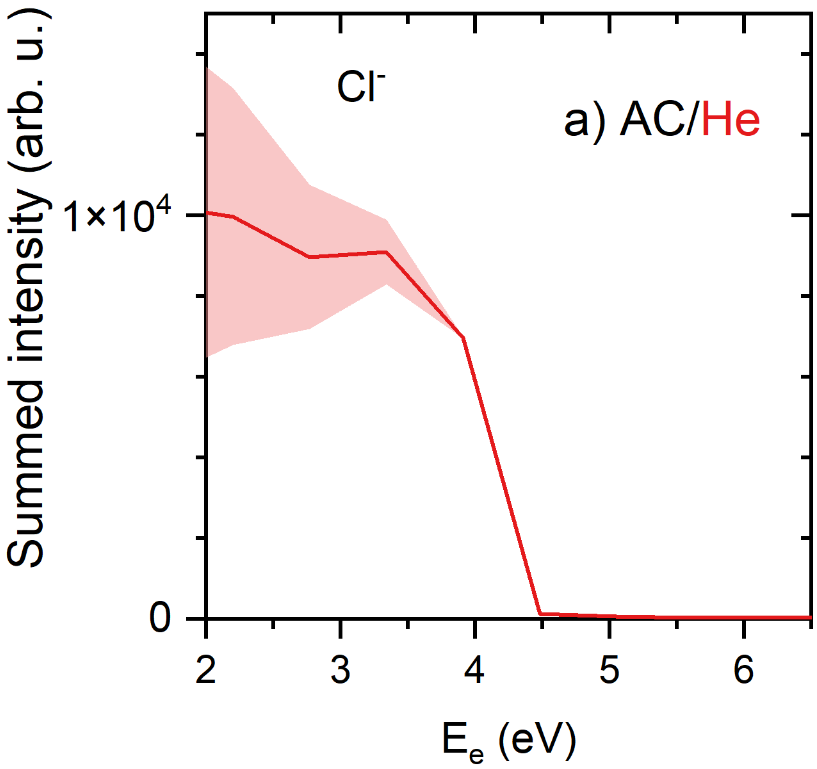

Fig. S3. Electron energy dependent yield of Cl^- fragment formed in molecular beam.

III. ANALYSIS OF THE AMBIGUOUS CLUSTER SERIES $AC_n{\cdot}X^-$

The mass peak multiplets denoted as $(AC)_nX^-$ in Fig. 4 in the main article could not be unambiguously assigned. Most likely, the starting mass could be assigned to the H-abstraction process; i.e., $[(AC)_n\text{-H}]^-$ (for $n = 2$ $m/z = 155,\ 157,\ 159$). The intensity of the mass peaks at higher masses exceeds the isotopologue contribution of the $[(AC)_n\text{-H}]^-$ ions. Thus, further ions have to contribute there. A simple possibility is the intact parent ion clusters $[(AC)_n]^-$ (for $n = 2$ $m/z = 156,\ 158,\ 160$). The cluster anions with an additional H-atom $[(AC)_n\text{+H}]^-$ (for $n = 2$ $m/z = 157,\ 159,\ 161$) are also mentioned in the main text.

However, other ions can be considered simply based on a combination of masses of atoms available in the clusters. Possible candidates are $(AC)_nClCOO^-$ with the same mass and isotopological distribution as $[(AC)_n\text{+H}]^-$ (and so, indistinguishable by our means); for the few last peaks, $(AC)_n(CH_3)_3HCl^-$ or $(AC)_n(CH_3CO)C_3H_2^-$ would suit. Although the

latter differ by their number of Cl atoms, the isotopologue ratios cannot be used to clearly distinguish between them, since their intensities in the spectra for $n \geq 1$ are already so low that fitting the spectra with both candidates yields approximately the same errors (see Fig. S4, panels b, c, and d).

An alternative possibility would be to consider $(AC)_nCH_3Cl_4^-$ series instead of deprotonated and protonated AC. The first member of this series for $n = 0$ would yield the peak at $m/z = 155$ assigned to $[(AC)_2\text{-H}]^-$ in the main article. However, this alternative assignment would yield a relatively intense peak at $m/z = 159$ (compare panels b and d in Fig. S4). Due to the presence of 4 Cl atoms in this ion, the third member at $m/z = 159$ of this multiplet, which starts at $m/z = 155$, has still about 50% abundance of the second peak at $m/z = 157$, which is the main one. However, even for clusters of $n = 2$, these two variants fit similarly, due to the low intensity of signal.

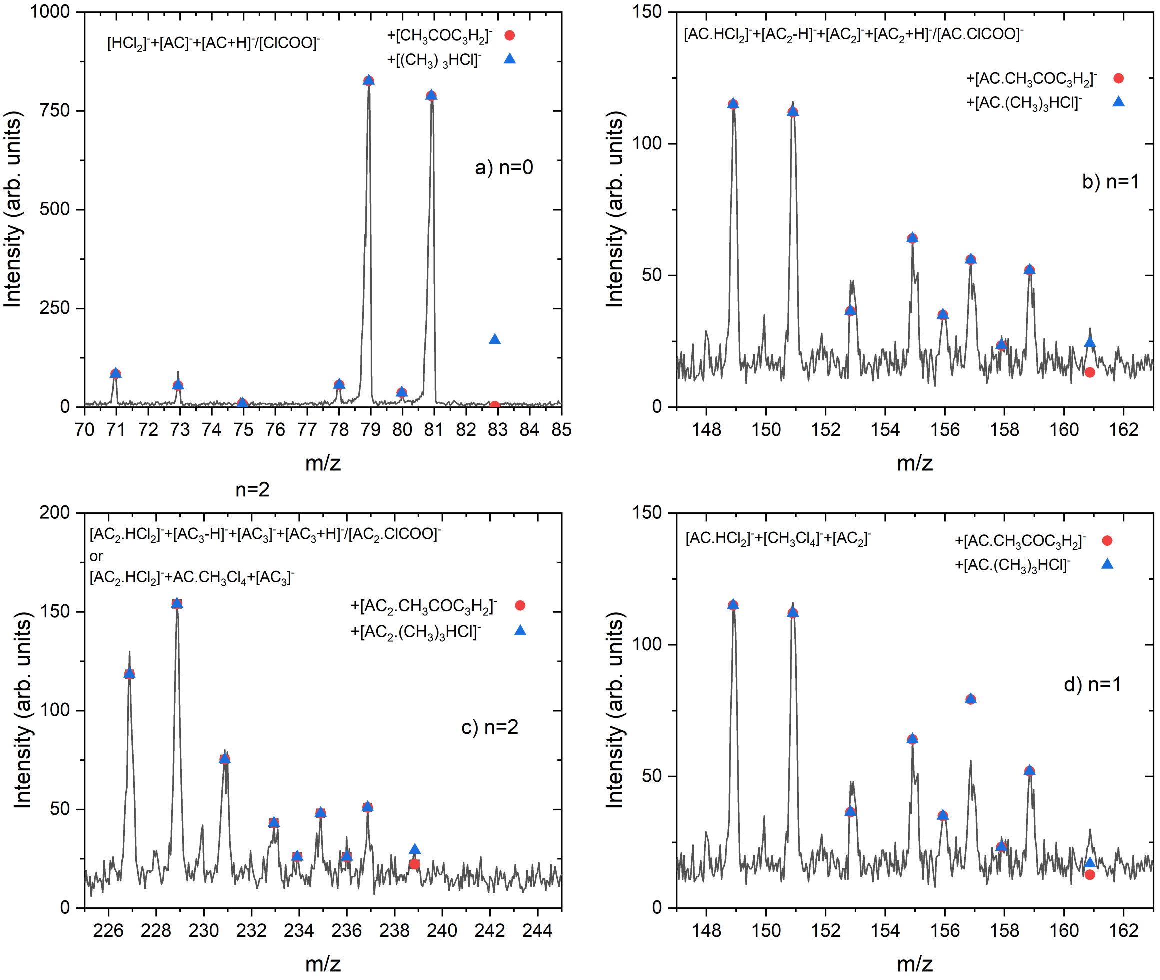

Fig. S4. Compilation of different fragments combinations for cluster size $n = 0$ (a), 1 (b) (d), 2 (c). Intensities marked by points are calculated as a sum of intensities of all denoted fragments. Fragment intensity is obtained by scaling the relative abundances (above 10%) of isotopologues to the monoisotopic peak maximum. Any lighter fragments overlapping with this peak are deducted from the peak maximum pre-scaling.

IV. COORDINATES OF ALL CALCULATED STRUCTURES

Cartesian coordinates (in Å) of all optimized molecules and ions, along with their relative energies (in Hartree), calculated at the ωB97XD/def2TZVP level of theory, are provided below.

AC, $E = -613.425496$

```
Cl  1.2883850 -0.1482650  0.0000000
O  -0.8284600  1.3630930  0.0000010
C  -1.3185140 -0.9909990 -0.0000020
C  -0.4708750  0.2415410 -0.0000050
H  -1.0851980 -1.5909850 -0.8795930
```

```
H  -2.3680110 -0.7056270 -0.0000850
H  -1.0853210 -1.5908670  0.8797050
```

$(\mathrm{AC})_2$, $E = -1226.85719$

```
C  -2.4230050  1.5071120 -0.3644120
C  -2.1218510  0.2033660  0.2948820
Cl -1.7465060 -1.0948100 -0.9078710
H  -3.2370540  1.3799200 -1.0774650
H  -2.6845260  2.2397190  0.3955220
H  -1.5376730  1.8310900 -0.9113270
O  -2.1048070 -0.0473400  1.4455780
H   0.3467840 -0.8311750  1.7111480
C   1.3065410 -0.9490460  1.2134240
H   2.1149690 -1.0289930  1.9399700
H   1.3083640 -1.8592060  0.6129910
O   0.8416000  1.1389390  0.1121440
C   1.5543350  0.2212930  0.3196490
Cl  3.1523100  0.1324280 -0.4988270
```

$(\mathrm{AC})_3$, $E = -1840.290566$

```
C   2.3474820  2.4259590 -0.1564370
C   0.9159660  2.1494020  0.1562070
Cl  0.0669950  1.3254670 -1.2121530
H   2.8584970  1.4833100 -0.3584340
H   2.8052520  2.9363960  0.6876630
H   2.4100000  3.0366570 -1.0571930
O   0.3203660  2.3988880  1.1419340
C  -1.9253500 -0.2184940  1.5563070
C  -2.4070060 -0.7664390  0.2553880
Cl -3.8684630  0.0825250 -0.3486200
H  -1.6344250  0.8241530  1.4197580
H  -1.0702810 -0.7985980  1.8962010
H  -2.7321950 -0.2492260  2.2882480
O  -1.9432120 -1.6517680 -0.3746500
C   1.3124890 -2.6038910 -0.8411440
C   2.2027600 -1.4978980 -0.3926610
Cl  1.9384200 -1.0734240  1.3508080
H   0.2714350 -2.2991410 -0.7184090
H   1.5243840 -2.8333950 -1.8827970
H   1.4762130 -3.4791460 -0.2123370
O   3.0084560 -0.9021830 -1.0121910
```

$(\mathrm{AC})_4$, $E = -2453.725443$

```
C  -3.0131490 -0.8761750 -0.6614620
C  -1.8565400 -1.6487690 -1.1971940
Cl -1.5970380 -3.1928570 -0.3150220
H  -2.8312700 -0.6528930  0.3898940
H  -3.1244410  0.0458660 -1.2271060
H  -3.9163380 -1.4833300 -0.7265850
O  -1.1463140 -1.3633800 -2.0972070
C   1.0842280  1.1455040  2.3373500
C  -0.0069770  0.3521840  1.7077560
Cl -1.3403690 -0.0270470  2.8503180
H   1.8575820  1.3448280  1.6003530
H   1.4926370  0.5858260  3.1787070
H   0.6702860  2.0805030  2.7153630
O  -0.0802800 -0.0283060  0.5899990
C   2.0594890 -2.1803470 -0.9469050
C   2.7030830 -1.2719530  0.0429490
Cl  3.3882320  0.2126440 -0.7377040
H   2.7750440 -2.4386890 -1.7271640
H   1.2220930 -1.6629790 -1.4164390
H   1.7022530 -3.0718920 -0.4373160
O   2.8057060 -1.4097390  1.2083390
C   0.1505460  1.8985820 -2.1963240
C  -0.1129290  2.6642130 -0.9456430
Cl -1.8871310  2.8206940 -0.6025050
H  -0.2840040  0.9023070 -2.1070760
H   1.2243650  1.8302120 -2.3533620
H  -0.3332030  2.3992570 -3.0353400
O   0.6603470  3.1505700 -0.2028280
```

$(\mathrm{AC})_5$, $E = -3067.158802$

```
C  -3.7602970 -1.1408020  1.1702020
C  -3.7377940 -0.7781600 -0.2763520
Cl -5.2018310  0.1279810 -0.7875090
H  -3.9258280 -0.2422380  1.7637110
H  -2.8176890 -1.6096300  1.4431370
H  -4.5969820 -1.8168580  1.3524150
O  -2.8980010 -1.0208230 -1.0714750
C   0.2885180 -2.5974890 -1.2972320
C   1.4154090 -1.8862300 -1.9625300
Cl  3.0175880 -2.6125140 -1.5658620
H   0.2962050 -3.6452970 -1.5988530
H  -0.6529520 -2.1235780 -1.5635360
H   0.4314990 -2.5633430 -0.2166420
O   1.3702020 -0.9508180 -2.6817800
C  -0.9461940  1.4774250 -1.9091800
C  -0.6948860  1.1548160 -0.4782070
Cl -1.8191910  2.0238570  0.6360500
H  -0.8126230  2.5492380 -2.0573130
H  -1.9767250  1.2233150 -2.1568260
```

H	-0.2526900	0.9110240	-2.5264400
O	0.1217630	0.4352340	-0.0207150
C	0.3477890	0.1439750	3.2452570
C	0.3863950	-1.1537000	2.5154440
Cl	2.0687700	-1.6897070	2.1571200
H	0.8584410	0.0297490	4.2023260
H	-0.6873440	0.4409910	3.3957020
H	0.8834740	0.8988530	2.6707050
O	-0.5180500	-1.8356020	2.1820610
C	3.3736090	1.0615050	-0.4546850
C	2.6649710	2.1442160	0.2838670
Cl	2.0639370	3.4446440	-0.8276540
H	2.7291720	0.6705740	-1.2411320
H	3.6467960	0.2718660	0.2410620
H	4.2618970	1.4782840	-0.9311150
O	2.4731570	2.2434200	1.4415130

$(\mathrm{AC})^-$, $E = -613.438898$

Cl	-2.3374430	-0.1896480	0.0003100
O	2.7009670	-0.6650690	0.1658180
C	0.8394290	0.8839610	0.0932360
C	1.9097080	-0.0070660	-0.4188340
H	-0.1454970	0.3619840	0.0175470
H	0.7558940	1.7703440	-0.5339350
H	1.0235830	1.1508640	1.1381690

$(\mathrm{AC})_2^-$, $E = -1226.887011$

C	-0.8802480	0.3022020	1.1422920
C	-2.1515680	-0.0963660	0.5081440
Cl	-1.8869690	-1.2017660	-0.9371180
H	-0.3749960	1.0771840	0.5221290
H	-1.0907780	0.7315040	2.1201620
H	-0.1939230	-0.5408330	1.2140660
O	-3.2602210	0.1984630	0.7807640
H	1.1285150	-0.9689320	-1.2380820
C	2.0707450	-0.6261030	-0.8108900
H	2.8910300	-0.7129850	-1.5290260
H	1.9082650	0.4367640	-0.5411170
O	3.3751850	-1.8736660	0.8097290
C	2.3652180	-1.3938650	0.4279090
Cl	1.0862210	2.6290430	-0.2909300

$(\mathrm{AC})_3^-$, $E = -1840.329912$

C	-2.2091110	0.1526130	0.6423890
C	-2.5572700	-1.1827040	0.0948670
Cl	-4.2801920	-1.2275210	-0.5674040
H	-2.0298730	0.8199130	-0.2062570
H	-1.3051060	0.1111330	1.2614920
H	-3.0435270	0.5606990	1.2084900
O	-1.9096770	-2.1591840	0.0070690
C	1.2390760	-0.6971980	-0.3148950
C	2.6371710	-0.6465310	-0.7938830
Cl	3.6429070	-2.0338770	-0.1601320
H	0.6228430	-0.0650290	-0.9544270
H	1.2029520	-0.2800630	0.7122790
H	0.8600710	-1.7149670	-0.2764010
O	3.1626470	0.1556810	-1.4822570
C	0.9993560	2.8599080	-0.2742520
C	-0.2318280	2.7124250	-1.0968750
Cl	0.5697290	0.9505080	2.6105800
H	0.8241280	2.3428330	0.6880970
H	1.8261590	2.3437290	-0.7624310
H	1.2315690	3.9149460	-0.1055050
O	-1.0411110	3.5111100	-1.4149590

$(\mathrm{AC})_4^-$, $E = -2453.774894$

C	2.9436050	0.4705440	-0.7352550
C	4.1819260	-0.2911650	-1.0226670
Cl	4.3895320	-1.7135700	0.1080600
H	2.8457700	0.6468990	0.3346330
H	2.0699030	-0.1195850	-1.0622460
H	2.9635610	1.4090600	-1.2854850
O	4.9990860	-0.1093190	-1.8516380
C	-3.1527250	-1.5416260	-0.1278670
C	-4.3818980	-0.9829410	-0.7382080
Cl	-4.4980350	0.8282720	-0.5127810
H	-2.2756550	-1.2485480	-0.7303280
H	-3.2239800	-2.6274060	-0.1142880
H	-3.0097180	-1.1480170	0.8768620
O	-5.2424960	-1.5308440	-1.3277660
C	0.8192880	-1.6466350	1.6487750
C	-0.3794400	-2.1349890	2.3883320
H	1.6405740	-2.3558020	1.7166120
H	1.1164260	-0.6595310	2.0132200
H	0.5257280	-1.5671790	0.5861910
O	-1.2449560	-1.5186080	2.9053940
C	-0.8847340	1.3948790	0.7147410
C	0.3183120	2.1776630	1.0760680
Cl	0.6223180	3.5571900	-0.0757410
H	-0.6223980	0.7469490	-0.1430370
H	-1.1691200	0.7676660	1.5585510
H	-1.7012320	2.0415150	0.4027940

```
O    1.0846090  2.0031960  1.9573420
Cl -0.0853330 -0.9845860 -1.6868110
```

$(\mathrm{AC})_5^-$, $E = -3067.216436$

```
C    2.6896950  1.8600660 -0.6997310
C    4.0236870  1.2127760 -0.6887100
Cl   4.1961640  0.0280490  0.6960850
H    2.4399180  2.2275680  0.2945110
H    1.9286170  1.1167640 -0.9873990
H    2.6907810  2.6709840 -1.4248890
O    4.9320760  1.3524990 -1.4242060
C   -3.0164970 -1.4839720 -0.3086830
C   -4.2525610 -1.1277080 -1.0478730
Cl  -4.7039640  0.6278930 -0.8179040
H   -2.1524650 -1.0026690 -0.7934470
H   -2.8836700 -2.5635010 -0.3320450
H   -3.0733530 -1.1193880  0.7159930
O   -4.9358840 -1.8001730 -1.7318130
C    0.5977630 -0.3251910  1.8016810
C   -0.6246760 -0.8863440  2.4219790
Cl  -0.6298100 -2.7091490  2.4261300
H    1.4831560 -0.8921430  2.0775310
H    0.6910070  0.7233750  2.0798280
H    0.4718650 -0.3960940  0.7059700
O   -1.5644620 -0.3215160  2.8581230
C   -1.5066660  2.2631160  0.3072430
C   -0.4192790  3.2092940  0.6501100
Cl  -0.1531890  4.4615590 -0.6418240
H   -1.0996210  1.5478620 -0.4288520
H   -1.8080380  1.7217920  1.2025500
H   -2.3481190  2.7747400 -0.1528400
O    0.2820460  3.2224080  1.6004080
Cl   0.0039340 -0.2178290 -1.6386270
O    2.7147180 -4.9324640 -1.9391920
C    1.8937080 -4.3568790 -1.3178970
C    1.9797290 -3.0431990 -0.6102740
H    1.3478440 -2.3155790 -1.1369590
H    3.0108150 -2.6830200 -0.5698520
H    1.5605990 -3.1473850  0.3906530
```

$(\mathrm{AC})\mathrm{Cl}^-$, $E = -1073.72149$

```
C   -0.0288940  0.6679150  0.9372550
C    1.1283290  0.6283640  0.0286150
Cl  -2.9769180 -0.2254150 -0.1973870
H    0.1306760  0.0416610  1.8111340
H   -0.9423290  0.2749410  0.4123150
H   -0.2309940  1.7030570  1.2081190
O    1.5965300  1.4763930 -0.6446100
Cl   1.8989060 -1.0456710 -0.0420190
```

$(\mathrm{AC})_2\mathrm{Cl}^-$, $E = -1687.169096$

```
C   -1.5901860  0.1862620 -1.1588820
C   -2.8286020 -0.2925120 -0.5126260
Cl  -2.4820500 -1.2495490  1.0176880
H   -0.8315120 -0.5944560 -1.1761250
H   -1.1741740  1.0394560 -0.5797980
H   -1.8267890  0.5344750 -2.1626590
O   -3.9550920 -0.1370980 -0.8233100
C    1.3856570 -0.1634740  1.0276040
C    2.0710400 -0.8238160 -0.1004440
Cl   3.8714770 -0.4683300 -0.1331930
H    1.1159570  0.8734300  0.7171130
H    2.0245040 -0.1009750  1.9041630
H    0.4557600 -0.6907500  1.2373510
O    1.6247720 -1.4988310 -0.9591050
Cl   0.0606530  2.8112620  0.2205260
```

$(\mathrm{AC})_3\mathrm{Cl}^-$, $E = -2300.613374$

```
C    0.0596790 -2.7158220  0.3116680
C   -0.9737210 -2.4243100 -0.7043440
Cl  -2.6319450 -3.0020150 -0.2002890
H   -0.0209340 -1.9377340  1.1006420
H    1.0440480 -2.6445080 -0.1487770
H   -0.1042430 -3.6855420  0.7749210
O   -0.8694520 -1.8587400 -1.7354330
Cl   0.0380350 -0.1016370  2.5130080
C    1.6822680  0.4269050 -0.4644650
C    2.9908880 -0.2629310 -0.5086720
Cl   4.3634590  0.8013550  0.0590010
H    1.2926540  0.3534930  0.5705750
H    0.9886740 -0.0874340 -1.1286270
H    1.7717620  1.4795390 -0.7179630
O    3.2387600 -1.3719290 -0.8270280
C   -2.1741650  0.9460670  0.0733090
C   -2.3861120  2.4050770 -0.0582270
Cl  -0.9704160  3.2329060 -0.8727020
H   -1.7846460  0.5304130 -0.8541810
H   -3.1127320  0.4712440  0.3523360
H   -1.4280420  0.7566380  0.8688340
O   -3.2974080  3.0673720  0.2878740
```

$(\mathrm{AC})_4\mathrm{Cl}^-$, $E = -2914.056169$

C	-3.1671150	-0.8386220	-0.7213650
C	-4.2922030	0.0251040	-1.1515600
Cl	-4.2587040	1.6556060	-0.3243330
H	-3.0690290	-0.8217820	0.3628920
H	-2.2276030	-0.4530340	-1.1530780
H	-3.3340730	-1.8512990	-1.0823560
O	-5.1528810	-0.1943660	-1.9254820
C	3.1671120	0.8385980	-0.7213360
C	4.2922070	-0.0251170	-1.1515370
Cl	4.2586870	-1.6556450	-0.3243630
H	2.2276070	0.4530230	-1.1530780
H	3.3340780	1.8512850	-1.0822960
H	3.0690070	0.8217270	0.3629180
O	5.1529010	0.1943760	-1.9254350
C	-0.6736520	1.6970810	0.9966290
C	0.6255620	2.1505060	1.5418850
Cl	1.1513450	3.7518960	0.8492210
H	-1.3732180	2.5223100	0.8921340
H	-1.0729520	0.9091570	1.6334520
H	-0.4819290	1.2833480	-0.0115970
O	1.3430400	1.6072870	2.3065480
C	0.6736500	-1.6970500	0.9966590
C	-0.6255670	-2.1504750	1.5419050
Cl	-1.1513290	-3.7518810	0.8492600
H	0.4819380	-1.2833350	-0.0115770
H	1.0729380	-0.9091130	1.6334730
H	1.3732240	-2.5222760	0.8921870
O	-1.3430600	-1.6072490	2.3065480
Cl	0.0000030	-0.0000080	-1.9537950

$(AC)HCl_2^-$, $E = -1534.569566$

H	2.4951140	0.1462560	-0.0399540
Cl	2.7651910	-1.3230460	-0.3791720
Cl	2.1589630	1.6656320	0.3176810
Cl	-2.8798480	0.4595080	-0.8752340
O	-2.6447770	-0.7393610	1.4272030
C	-0.5694810	-0.5887800	0.2054000
C	-2.0136000	-0.4139720	0.4868810
H	-0.4110610	-0.9945840	-0.7917380
H	-0.1211130	-1.2379170	0.9531930
H	-0.0594500	0.3820450	0.2315200

$(AC)_2HCl_2^-$, $E = -2148.012815$

C	1.6698090	0.1118830	0.8277360
C	2.5699930	-0.9966210	0.4255180
Cl	1.8458620	-2.0074380	-0.9136960
H	1.5049400	0.7846110	-0.0207430
H	2.1092970	0.6655190	1.6534660
H	0.6949900	-0.2831500	1.1139630
O	3.6359330	-1.2873880	0.8359270
C	-1.6628220	0.1304430	-0.8295710
C	-2.5795350	-0.9651290	-0.4291160
Cl	-1.8794070	-1.9764680	0.9221840
H	-1.4925930	0.8028860	0.0179480
H	-2.0905950	0.6876740	-1.6590180
H	-0.6921550	-0.2786690	-1.1103700
O	-3.6450510	-1.2458450	-0.8476190
Cl	0.6108670	2.7353470	-1.4441420
H	0.0178840	2.7870970	0.0002340
Cl	-0.5751750	2.7436430	1.4433390

$(AC)_3HCl_2^-$, $E = -2761.447446$

C	1.7817020	1.6881130	-0.2945250
C	0.4051560	1.7952780	-0.8394270
Cl	-0.5673760	3.1063860	0.0014800
H	2.2599910	2.6662830	-0.2839300
H	2.3694080	0.9831000	-0.8831720
H	1.7309860	1.3481460	0.7410430
O	-0.1177360	1.1756530	-1.6920800
C	1.9169580	-1.9067260	0.6982960
C	0.4590770	-1.6397670	0.6722730
Cl	-0.4631040	-2.8897200	-0.2820810
H	2.1059770	-2.9543950	0.9253980
H	2.4067970	-1.2467160	1.4132720
H	2.3435090	-1.7016810	-0.2905160
O	-0.1442780	-0.7513310	1.1617100
C	-2.9580710	0.0914140	-0.6001650
C	-4.3719500	-0.3310810	-0.4153920
Cl	-5.0608990	0.2685440	1.1610130
H	-2.8868150	1.1783480	-0.5553040
H	-2.5882810	-0.2719020	-1.5552760
H	-2.3437220	-0.3023020	0.2108070
O	-5.0573560	-0.9684210	-1.1302530
Cl	4.5416930	0.4701430	1.5661090
H	4.5303170	-0.0373740	0.1337810
Cl	4.4455440	-0.5725360	-1.3816680

$[(AC)_2{-}H]^-$, $E = -1226.316964$

C	1.0700990	0.3800950	1.1400440
C	2.2812410	-0.1404210	0.4774190
Cl	1.8844430	-1.3922770	-0.8080510
H	0.5570220	1.1106730	0.4738310

```
H   1.3588790  0.8938870  2.0552790
H   0.3502700 -0.4135560  1.3290870
O   3.4149030  0.1393920  0.6436480
C  -1.9871280 -0.6608130 -0.4190000
C  -3.0623700 -1.1390140  0.1294180
Cl -0.9481600  2.5709910 -0.4083210
H  -1.7862070  0.4248700 -0.4162120
H  -1.2763860 -1.3377620 -0.8706570
O  -4.0313310 -1.5588080  0.6238150
```

$[(\mathrm{AC})_3{-}\mathrm{H}]^-$, $E = -1839.76169$

```
C   2.6100820  0.2270130  0.2986320
C   2.3211040 -0.7713120 -0.7541930
Cl  2.5680180 -2.4861830 -0.1600850
H   1.7868900  0.2233910  1.0423640
H   2.6749330  1.2130030 -0.1576890
H   3.5236410 -0.0296290  0.8312310
O   1.9511090 -0.6116660 -1.8618360
Cl  0.1451080  0.5988400  2.6573510
C  -0.3851280  2.5104300 -0.1446660
C   0.5699700  3.2394080 -0.6384720
H  -0.2390970  2.0129740  0.8249140
H  -1.3005590  2.3743920 -0.7012130
O   1.4388710  3.8872980 -1.0644450
C  -1.1460630 -1.0455030 -0.0881380
C  -2.5997220 -1.2887250 -0.2142940
Cl -3.5203840  0.2380840 -0.6473950
H  -0.7878850 -0.4049890 -0.8919210
H  -0.6242610 -2.0009440 -0.0792940
H  -0.9418420 -0.5176450  0.8628910
O  -3.2137180 -2.2832600 -0.0652570
```

$[(\mathrm{AC})_4{-}\mathrm{H}]^-$, $E = -2453.205407$

```
C   3.3252040 -0.1795170  1.4639470
C   3.9612030  0.9191840  1.1815190
H   2.3201320 -0.1177530  1.8949850
H   3.7763590 -1.1301260  1.2219120
O   4.5178620  1.9097650  0.9348280
C  -2.8516510  0.4127870  0.3169520
C  -3.9021830 -0.6154970  0.5025720
Cl -3.4271710 -2.2205450 -0.2354660
H  -1.9958850  0.1808550  0.9751470
H  -3.2520550  1.3875180  0.5882290
H  -2.4943080  0.4098520 -0.7113580
O  -4.9444350 -0.5345290  1.0459090
C   1.0492860  1.9838640 -0.4318370
C  -0.1499920  2.3162310 -1.2336440
Cl -0.9827800  3.8284980 -0.6486710
H   1.6162040  2.8764960 -0.1785830
H   1.6589040  1.2645110 -0.9763220
H   0.6982410  1.5186340  0.5091620
O  -0.6277450  1.7265810 -2.1379900
C   0.3548750 -1.5034140 -0.7460180
C   1.7310560 -1.7903960 -1.2136290
Cl  2.4038070 -3.3262400 -0.4777540
H   0.3953410 -1.1148840  0.2866140
H  -0.0856210 -0.7461770 -1.3928730
H  -0.2493950 -2.4075530 -0.7288570
O   2.4140050 -1.1784230 -1.9534530
Cl -0.0157520  0.1437000  2.3241490
```

$[(\mathrm{AC})_2{-}\mathrm{H}]^-$ (m), $E = -1226.359085$

```
C  -1.3175360  1.7379170  0.0000270
C  -0.4590310  0.5078300  0.0000020
Cl  3.5987730  0.1955740  0.0002150
H  -0.7099230  2.6408430  0.0000940
H  -1.9784640  1.7212920  0.8676380
H  -1.9784070  1.7213760 -0.8676280
O  -1.0455860 -0.6418360 -0.0000770
C   0.9103030  0.6402760  0.0000770
C   1.8076710 -0.4611970  0.0000770
Cl -3.9120230 -0.4003980 -0.0002540
H  -2.0831340 -0.5690460 -0.0001420
H   1.3350110  1.6288620  0.0001490
O   1.6820510 -1.6344480  0.0000090
```

$[(\mathrm{AC})_3{-}\mathrm{H}]^-$ (m), $E = -1839.800969$

```
C  -2.1562970  2.7161270  0.3104050
C  -1.8652320  1.2624530  0.0900390
Cl -4.3293680 -1.9588220  0.0447120
H  -3.2174380  2.8846590  0.4821110
H  -1.8159580  3.2908780 -0.5522820
H  -1.5726040  3.0796620  1.1575020
O  -0.6359640  0.9228060 -0.1286110
C   2.1732160 -0.3605840 -0.6811630
C   3.4559490 -1.0916800 -0.5389690
Cl  3.9071260 -1.3520860  1.2118650
H   1.3861620 -0.8409780 -0.1013440
H   1.8974380 -0.3213700 -1.7324080
H   2.2787810  0.6613280 -0.2977980
O   4.1848770 -1.4958040 -1.3728040
C  -2.8821480  0.3424310  0.1227600
```

C	-2.6751710	-1.0530700	-0.0722840
Cl	1.2445040	3.0911750	-0.0960260
H	0.0190840	1.7249640	-0.1152490
H	-3.8871070	0.6812520	0.3053870
O	-1.7200040	-1.7168760	-0.2810870

$[(AC)_4{-}H]^-$ (m), $E = -2453.243639$

C	2.3297250	0.7526980	2.2408860
C	1.7287290	-0.3612880	1.4435470
Cl	3.3355300	-2.9808980	-1.1852060
H	3.3928620	0.8570200	2.0366380
H	2.1682460	0.5672820	3.3044030
H	1.8108600	1.6841870	2.0080040
O	0.4566000	-0.5834020	1.5858520
C	-2.4288560	-1.1644150	0.6979720
C	-3.7173480	-1.3442920	-0.0156980
Cl	-3.8235390	-0.3001930	-1.5128470
H	-1.5881130	-1.3458200	0.0286040
H	-2.3856520	-1.8519300	1.5393590
H	-2.3430380	-0.1369150	1.0675270
O	-4.6330940	-2.0353390	0.2517610
C	2.4993520	-1.1175880	0.6022450
C	1.9605770	-2.1408140	-0.2335900
Cl	-1.0448160	1.6101890	2.6850450
H	-0.0345900	0.1716070	2.0804050
H	3.5562470	-0.9230880	0.5446420
O	0.8574330	-2.5239780	-0.4321970
C	-0.3298950	1.4881450	-0.8755810
C	0.3718410	2.5978570	-1.5679050
H	0.2870530	0.5897880	-0.8796170
H	-1.2848030	1.3044780	-1.3639380
H	-0.4992240	1.7618350	0.1745830
Cl	2.0694880	2.8295360	-0.9387280
O	-0.0169220	3.3130870	-2.4194610

CH_3CO, $E = -153.147093$

O	1.2550340	0.1730630	0.0000020
C	-1.1643450	0.0976670	-0.0000140
C	0.2467380	-0.4292450	-0.0000180
H	-1.6786670	-0.2929230	-0.8783050
H	-1.1778580	1.1898040	-0.0005900
H	-1.6781100	-0.2919180	0.8790660

HCl, $E = -460.810292$

H	0.0000000	0.0000000	-1.2072750
Cl	0.0000000	0.0000000	0.0710160

H_2CCO, $E = -152.610862$

O	0.0000000	0.0000000	1.2582850
C	0.0000000	0.0000000	0.1034250
C	0.0000000	0.0000000	-1.2035960
H	0.0000000	0.9398880	-1.7326270
H	0.0000000	-0.9398880	-1.7326270

Cl^-, $E = -460.269794$

Cl	0.0000000	0.0000000	0.0000000

$[(AC)_2{-}Cl]$, $E = -766.577577$

C	-1.3030560	1.6987200	-0.0692070
C	-1.2169230	0.3113370	0.4760730
Cl	-1.4872840	-0.9367230	-0.8031100
H	-2.2791500	1.8483690	-0.5309450
H	-1.1466720	2.4108210	0.7378140
H	-0.5411410	1.8242340	-0.8383470
O	-1.0138650	-0.0250200	1.5865600
H	1.8030300	-0.8718590	1.6480050
C	2.1199250	-1.0429230	0.6191720
H	3.1570730	-1.3820330	0.5789970
H	1.4481370	-1.7928830	0.2002670
O	2.7184160	0.8613250	-0.7604270
C	1.9344100	0.2323980	-0.1513680

$[(AC)_3{-}Cl]$, $E = -1380.010038$

C	-3.2074110	-1.5768550	-0.1054710
C	-1.7441610	-1.5386720	0.1812720
Cl	-0.7899030	-0.9187570	-1.2234460
H	-3.5511190	-0.5573140	-0.2816020
H	-3.7275900	-2.0072610	0.7470040
H	-3.3906800	-2.1599340	-1.0074550
O	-1.1859410	-1.8512040	1.1705240
C	1.5131630	0.2732560	1.4952650
C	2.2220910	0.4733960	0.1980640
Cl	3.4165710	-0.8263040	-0.1369440
H	0.8290240	1.1030540	1.6664690
H	2.2417630	0.2012740	2.3026910
H	0.9582370	-0.6654610	1.4596680
O	2.0786280	1.3500010	-0.5801210
C	-1.0266150	2.8621940	-0.6608650
C	-1.5198400	2.2047630	0.5917540
H	-0.9279240	3.9317020	-0.4718120
H	-0.0291380	2.4760260	-0.8764330

H	-1.7062520	2.6774580	-1.4956080
O	-2.4893170	1.5609540	0.7700460

$[(\mathrm{AC})_4\mathrm{-Cl}]$, $E = -1993.44312$

C	4.0253660	0.2407290	-0.1391580
C	3.1970420	-0.9997840	-0.1414630
Cl	2.1484080	-1.1084170	-1.6123200
H	3.3651680	1.1069580	-0.1753130
H	4.6543820	0.2570520	-1.0293980
H	4.6341440	0.2620910	0.7617510
O	3.1686400	-1.8630920	0.6585240
C	-4.0263240	-1.2076880	-0.9251970
C	-3.2534400	0.0640370	-0.8221300
Cl	-1.5687260	-0.1105540	-1.4510470
H	-4.0497820	-1.5359910	-1.9642710
H	-5.0335050	-1.0466070	-0.5482240
H	-3.5178710	-1.9761730	-0.3406310
O	-3.6117980	1.1035250	-0.3983200
C	-0.0382610	-2.3876310	1.4694860
C	-1.4934640	-2.0424310	1.4750770
H	0.0808920	-3.4453410	1.7020160
H	0.5212640	-1.7620590	2.1660360
H	0.3358610	-2.2338670	0.4558410
O	-2.0646520	-1.1797080	2.0390980
C	-0.9456100	2.0808080	1.5314900
C	0.3969530	1.7050700	1.0075310
Cl	1.0225380	2.8930030	-0.1990890
H	-1.6460320	2.1845180	0.7025840
H	-1.2900340	1.3119310	2.2191370
H	-0.8742310	3.0475960	2.0313740
O	1.0588650	0.7708620	1.2940760

$[(\mathrm{AC})_5\mathrm{-Cl}]$, $E = -2606.873272$

C	3.7317290	1.2434280	-0.4635160
C	4.6028050	0.0533140	-0.6670150
Cl	3.6669450	-1.5104410	-0.4902630
H	3.2769500	1.2058400	0.5267260
H	2.9164350	1.2299680	-1.1869460
H	4.3263550	2.1468430	-0.5762800
O	5.7492930	0.0015200	-0.9119770
C	-3.2009530	-0.7054620	-1.1435850
C	-4.5676060	-0.1407860	-0.9329790
Cl	-4.5107870	1.5192060	-0.2172310
H	-2.6124620	-0.0203120	-1.7538460
H	-3.2893070	-1.6766770	-1.6259590
H	-2.7041400	-0.8081700	-0.1770580
O	-5.6057670	-0.6407660	-1.1689590
C	0.8201290	-1.6468300	2.3116910
C	-0.5526050	-1.8142290	1.7606250
Cl	-0.7649900	-3.3938880	0.9073080
H	1.0729230	-2.5013700	2.9386030
H	0.8725810	-0.7139580	2.8662340
H	1.5265420	-1.6185930	1.4798860
O	-1.4642790	-1.0674630	1.8222150
C	-1.0698580	2.2514920	0.9300010
C	0.4120380	2.3138780	1.0713530
Cl	1.1293300	3.7112360	0.1892910
H	-1.3268190	2.1997260	-0.1271180
H	-1.4473650	1.3780560	1.4561500
H	-1.5100810	3.1644930	1.3320300
O	1.1183970	1.5871090	1.6778620
O	0.3695290	0.4626770	-1.4917240
C	0.3243990	-0.6933750	-1.2717330
C	0.3048810	-1.8408910	-2.2358910
H	1.1063800	-2.5299610	-1.9721730
H	0.4130030	-1.4924540	-3.2649930
H	-0.6365970	-2.3752190	-2.1030670

$(\mathrm{AC})\mathrm{HCl}$, $E = -1074.238983$

H	-1.6896560	-0.5233820	-0.0000050
Cl	-2.7941310	0.1373250	0.0000300
Cl	0.6869030	-1.2317470	-0.0000370
O	2.7827880	0.3155530	0.0001000
C	0.6983850	1.5237230	-0.0000750
C	1.6096450	0.3419360	0.0000090
H	0.0536140	1.4885350	-0.8783950
H	1.2948520	2.4330340	-0.0001030
H	0.0535810	1.4886200	0.8782240

$(\mathrm{AC})_2\mathrm{HCl}$, $E = -1687.6692$

C	1.9924260	0.5939530	1.2799810
C	2.6873720	-0.2639930	0.2760050
Cl	1.5518380	-0.8573170	-1.0106950
H	1.5695480	1.4641150	0.7758900
H	2.7049210	0.9028220	2.0413820
H	1.1675760	0.0378710	1.7258830
O	3.8205170	-0.5718980	0.2333280
C	-2.1389030	-0.6530790	-1.3866340
C	-1.9391560	-1.3394720	-0.0787250
Cl	-1.8756950	-0.1382350	1.3119450
H	-3.0530770	-0.0606270	-1.3507780
H	-2.1957650	-1.4007950	-2.1742710

```
H  -1.3052430  0.0260000 -1.5682860
O  -1.8355310 -2.4812110  0.1593080
Cl -0.6872490  2.8496290 -0.4990700
H  -1.1894920  1.9117100  0.2282760
```

$(AC)_3HCl$, $E = -2301.105974$

```
C  -3.4242100 -0.4805520  0.9546480
C  -2.5224460 -1.1687530 -0.0117460
Cl -2.0584540 -2.8078360  0.5134620
H  -4.3292490 -1.0738260  1.0879430
H  -3.6660510  0.5131890  0.5845900
H  -2.9182300 -0.4111960  1.9167960
O  -2.1138060 -0.7541290 -1.0453330
C   0.5930210  2.6930380  0.7358590
C   0.4551660  1.3282150  1.3174260
Cl  1.9415250  0.7880040  2.1775200
H   0.8465300  3.4005310  1.5254450
H  -0.3378970  2.9690650  0.2454620
H   1.4098710  2.6830540  0.0140620
O  -0.4787260  0.6064430  1.2717120
C   1.3436810 -1.6433740 -0.8302140
C   2.2249580 -0.4821800 -1.1499640
Cl  3.9719040 -0.8787230 -1.0110790
H   1.6757390 -2.5308900 -1.3660430
H   0.3152530 -1.3960270 -1.0824800
H   1.4227110 -1.8482740  0.2393400
O   1.8938200  0.6111910 -1.4488710
Cl -2.5932790  2.3911290 -1.5635590
H  -2.2988190  1.1342200 -1.4590740
```

$[(AC)_2{-}HCl]$, $E = -766.007554$

```
C   0.7457620  1.4905990  0.1409880
C   1.7150710  0.3557320  0.0933400
Cl  0.9044730 -1.2104410 -0.3220230
H   0.2363700  1.5769520 -0.8188400
H   1.2793800  2.4082970  0.3772270
H  -0.0144360  1.2824610  0.8957320
O   2.8746490  0.3776210  0.2876120
C  -2.4757770 -0.4218230  1.1652310
C  -2.4866180  0.0919370 -0.0365520
H  -1.7541240 -1.1895330  1.3979600
H  -3.2133040 -0.1020300  1.8845060
O  -2.4872190  0.5602150 -1.0926420
```

$[(AC)_3{-}HCl]$, $E = -1379.439842$

```
C  -2.8210250 -0.2481350  0.8984750
C  -1.5024380 -0.9230040  0.7069340
Cl -1.5305470 -2.1223730 -0.6267780
H  -3.1669340  0.1537090 -0.0521460
H  -2.7146940  0.5443490  1.6356840
H  -3.5487670 -0.9873650  1.2369040
O  -0.5159570 -0.7617780  1.3349470
C  -0.9174980  1.6520250 -1.5843520
C  -1.1299000  2.3941430 -0.5295860
H  -1.6747720  1.5991830 -2.3510460
H   0.0346880  1.1557120 -1.6948420
O  -1.3477630  3.0340810  0.4074980
C   2.7049690 -0.4398360  1.5628720
C   2.1856700  0.3299540  0.3953120
Cl  2.2501360 -0.6298550 -1.1331570
H   2.0858150 -1.3262730  1.6960850
H   2.6632120  0.1868340  2.4505600
H   3.7267730 -0.7621290  1.3632820
O   1.7690960  1.4318190  0.3746160
```

$[(AC)_4{-}HCl]$, $E = -1992.875075$

```
C  -1.7977880  1.5558860  1.5244110
C  -1.1940890  0.6955170  2.2993820
H  -1.2005880  2.2295820  0.9277550
H  -2.8766020  1.5814710  1.5044170
O  -0.6601010 -0.0681990  2.9840160
C   2.7337610  1.8536880 -1.6590620
C   1.5509100  2.0338030 -0.7692570
Cl  1.9290360  1.6416300  0.9537370
H   3.5606760  2.4619750 -1.2931830
H   2.4644200  2.1370740 -2.6737690
H   3.0426810  0.8083900 -1.6273120
O   0.4644690  2.3882390 -1.0586970
C   0.8457950 -2.2763310  0.7429090
C   1.6843790 -1.6912750 -0.3393290
Cl  3.4478920 -1.8866370 -0.0397170
H   1.1320330 -3.3141170  0.9110520
H  -0.2056020 -2.1997810  0.4731210
H   1.0334070 -1.7268490  1.6666890
O   1.3263210 -1.1311480 -1.3172940
C  -1.9708510 -0.0413310 -1.6996450
C  -2.8096420 -0.7870840 -0.7186870
Cl -4.4862580 -0.1253360 -0.6088670
H  -1.8914870  0.9980380 -1.3815490
H  -0.9812380 -0.4904700 -1.7471900
H  -2.4541350 -0.0580170 -2.6766470
```

```
O  -2.5081650 -1.7124800 -0.0524410
```

[(AC)$_2$−HCl] (m), $E = -766.022843$

```
C  -2.6913210 -1.0868650  0.0000400
C  -1.5479500 -0.1246850  0.0000540
Cl  2.4370610 -0.5504320 -0.0001290
H  -2.3449310 -2.1169020 -0.0000340
H  -3.3136430 -0.9285590 -0.8848390
H  -3.3135770 -0.9286630  0.8849840
O  -1.8674340  1.1707400  0.0001440
C  -0.2640790 -0.5309240 -0.0000210
C   0.8675500  0.3770170 -0.0000110
H  -2.8210210  1.2796700  0.0001870
H  -0.0580760 -1.5882200 -0.0000920
O   0.8969350  1.5583540  0.0000580
```

[(AC)$_3$−HCl] (m), $E = -1379.45935$

```
C   0.2105330  2.7042780  0.3652260
C  -0.3688720  1.4659380 -0.2309990
Cl -3.7400620 -0.6181880  0.3390610
H  -0.4518040  3.1332500  1.1122530
H   0.3927980  3.4480220 -0.4146160
H   1.1643160  2.4629270  0.8424990
O   0.3516500  0.8791010 -1.1885760
C   1.3474910 -1.9899080  0.2965070
C   2.0647250 -0.7527610  0.7096270
Cl  3.3827960 -0.2959870 -0.4609240
H   0.7600040 -1.7583480 -0.5949510
H   0.6811870 -2.2997750  1.0984550
H   2.0575280 -2.7766220  0.0478250
O   1.8682950 -0.0609580  1.6417460
C  -1.5478750  0.9465280  0.1658090
C  -2.0954860 -0.2875760 -0.3565200
H   1.1953110  1.3266790 -1.3031170
H  -2.1122160  1.4669060  0.9212380
O  -1.6295330 -1.0657770 -1.1201480
```

[(AC)$_4$−HCl] (m), $E = -1992.892098$

```
C  -0.9289850 -1.2192750  2.8210300
C  -1.2149730 -0.3841330  1.6185600
Cl -4.3487920  0.0263750 -0.8387490
H  -1.7922780 -1.8150460  3.1052660
H  -0.6485650 -0.5783390  3.6603140
H  -0.0907580 -1.8882010  2.6106050
O  -0.2377100  0.4187290  1.2060100
C   0.6645610  3.0498960 -0.8009420
C   1.6809410  1.9714530 -0.6564470
Cl  2.5584650  2.0687520  0.9378900
H  -0.1300900  2.8606700 -0.0784800
H   0.2503910  3.0179340 -1.8055280
H   1.1120970  4.0187450 -0.5848980
O   1.9537530  1.1081080 -1.4068920
C  -2.3989400 -0.4207890  0.9707610
C  -2.6757620  0.3475880 -0.2227580
H   0.5899220  0.2355130  1.6648080
H  -3.1772820 -1.0612370  1.3504580
O  -1.9807780  1.1043850 -0.8179930
C   0.2302340 -1.6928850 -1.4463800
C   1.6430000 -2.0932760 -1.1922000
H  -0.4395090 -2.3301500 -0.8687330
H   0.0225630 -1.7822530 -2.5099830
H   0.0806760 -0.6640510 -1.1203390
Cl  2.0614100 -2.0302940  0.5938800
O   2.4664800 -2.4396250 -1.9504480
```

[(AC)$_2$+H]$^-$ (v1), $E = -1227.553967$

```
C   2.5218270 -0.6190170  0.2606170
C   3.5782650  0.1176750 -0.3795810
Cl -2.0267520  1.6460910  0.1955540
H   2.7835540 -1.5551430  0.7356340
H  -2.8374360  0.0317430 -0.1769620
H   3.2706290  1.0691680 -0.8536530
O   4.7461790 -0.2320820 -0.4260340
C   0.1583560 -1.0022570  0.9730100
C   1.2273320 -0.2038690  0.2982930
Cl -3.4141830 -1.1999060 -0.4485830
H   0.5778010 -1.8806920  1.4593130
H  -0.3661990 -0.3841810  1.7039760
H  -0.5953700 -1.3137390  0.2461290
O   0.8604600  0.9271130 -0.2733440
H  -0.1048840  1.1322540 -0.1319440
```

[(AC)$_2$+H]$^-$ (v2), $E = -1227.539568$

```
C  -1.9538790 -0.5046110 -1.3784150
C  -0.7824210 -0.2740720 -0.4500530
Cl -1.2619920 -1.2044160  1.1787150
H  -2.8874400 -0.1726090 -0.9326900
H  -1.7794670  0.0573550 -2.2969080
H  -2.0050920 -1.5667590 -1.6115100
O   0.3225280 -0.7797690 -0.9881770
C   0.4134170  1.5756460  0.9747030
C  -0.6950660  1.1936990  0.0472230
```

```
Cl  3.0840640 -0.2270860 -0.1545140
H   0.4278100  0.9152570  1.8412760
H   0.2777660  2.6121090  1.2778180
H   1.3850700  1.4313890  0.4862930
O  -1.5109570  1.9874660 -0.3572190
H   1.2212660 -0.5467560 -0.5732860
```

(AC)ClCOO$^-$, $E = -1262.327121$

```
C  -0.0361990 -0.1688640  0.4942170
C  -1.5085650 -0.3899180  0.6173620
Cl  3.1423080 -0.9433450 -0.0226010
H   0.4147120 -0.2801490  1.4811890
H   0.4305240 -0.9135780 -0.1514930
H   2.1188710  0.6206380 -0.2301160
O  -2.1684160 -0.3895780  1.5933080
C   0.2769360  1.2412940 -0.0187520
Cl -2.2938220 -0.7480000 -0.9791990
O   1.5243810  1.4523900 -0.3342380
O  -0.5786390  2.0910490 -0.0873110
```

(AC)$_3^-$ (TS), $E = -1840.341733$

```
C  -4.5344310 -0.3005470 -0.5484050
C  -4.9893210 -1.6630410 -0.4396260
Cl -0.0977570  0.7000160  1.7791390
H  -5.1797260  0.4000060 -1.0613170
H   0.4056010  1.7811120  0.3694660
H  -4.3040320 -2.3508460  0.0910260
O  -6.0457180 -2.0806140 -0.8810830
C  -2.9159260  1.5660100 -0.1803180
C  -3.3538920  0.1429520 -0.0460960
Cl  0.7943110  2.5531370 -0.7126220
H  -3.6848760  2.1657060 -0.6627280
H  -2.6823440  1.9815770  0.8017590
H  -1.9937500  1.6229820 -0.7631490
O  -2.5304640 -0.6804760  0.5810110
H  -1.7307770 -0.2162290  0.9374380
C   4.0792310 -1.1216540  0.2299600
Cl  5.2260510 -0.7453590 -1.1368030
O   4.3666260 -1.9880630  0.9997470
C   2.9182890 -0.2990040  0.2213480
H   2.7709030  0.4639990 -0.5283390
H   2.1674600 -0.4358850  0.9921290
```

[AC-H], $E = -612.772045$

```
Cl  1.2642800  0.0772480  0.0000020
O  -0.9856320 -1.2467880 -0.0000030
C  -1.2425540  1.0828000  0.0000030
C  -0.5156290 -0.1495000 -0.0000020
H  -0.7363220  2.0353920 -0.0000400
H  -2.3222950  1.0259050  0.0000280
```

V. PARAMETERS OF GENETIC ALGORITHM RUNS

TABLE I. Parameters of genetic algorithm runs for different structures: number of structures in the initial population, side length of the cuboid box, total number of genetic algorithm cycles, number of best structures surviving each cycle, number of crossover operations per cycle, and mutation probability for each mutation function.

**Structure**	**Initial population**		**Genetic algorithm**			
	Num. structures	Box side length (Å)	Num. cycles	Num. best	Num. crossovers	Mutation prob. (%)
$(AC)_2$	200	12	50	50	50	20
$(AC)_3$	200	12	50	50	50	20
$(AC)_4$	200	14	52	50	50	20
$(AC)_5$	1000	20	64	100	50	20
$(AC)_2Cl^-$	200	16	66	50	50	20
$(AC)_3Cl^-$	200	17	111	50	50	20
$(AC)_4Cl^-$	1000	19	51	100	50	20
$(AC)HCl_2^-$	200	16	25	50	50	20
$(AC)_2HCl_2^-$	200	16	38	50	50	20
$(AC)_3HCl_2^-$	200	18	48	50	50	20
$[(AC)-H]^-$	200	8	50	50	100	25
$[(AC)_2-H]^-$	1000	12	50	250	250	25
$[(AC)_3-H]^-$	1000	14	99	250	250	25
$[(AC)_4-H]^-$	5000	17	99	250	250	25
$[(AC)_2+H]^-$	1000	10	25	250	250	25
